# Atg11 tethers Atg9 vesicles to initiate selective autophagy

**DOI:** 10.1371/journal.pbio.3000377

**Published:** 2019-07-29

**Authors:** Nena Matscheko, Peter Mayrhofer, Yijian Rao, Viola Beier, Thomas Wollert

**Affiliations:** 1 Molecular Membrane and Organelle Biology, Max Planck Institute of Biochemistry, Martinsried, Germany; 2 Membrane Biochemistry and Transport, Institut Pasteur Paris, Paris, France; The Francis Crick Institute, UNITED KINGDOM

## Abstract

Autophagy recycles cytoplasmic components by sequestering them in double membrane–surrounded autophagosomes. The two proteins Atg11 and Atg17 are scaffolding components of the Atg1 kinase complex. Atg17 recruits and tethers Atg9-donor vesicles, and the corresponding Atg1 kinase complex induces the formation of nonselective autophagosomes. Atg11 initiates selective autophagy and coordinates the switch to nonselective autophagy by recruiting Atg17. The molecular function of Atg11 remained, however, less well understood. Here, we demonstrate that Atg11 is activated by cargo through a direct interaction with autophagy receptors. Activated Atg11 dimerizes and tethers Atg9 vesicles, which leads to the nucleation of phagophores in direct vicinity of cargo. Starvation reciprocally regulates the activity of both tethering factors by initiating the degradation of Atg11 while Atg17 is activated. This allows Atg17 to sequester and tether Atg9 vesicles independent of cargo to nucleate nonselective phagophores. Our data reveal insights into the molecular mechanisms governing cargo selection and specificity in autophagy.

## Abstract 2

How do cells switch between selective and non-selective autophagy? This study shows that yeast Atg11 is a key coordinator of selective autophagy, binding and tethering donor vesicles to initiate the formation of selective autophagosomes. On induction of non-selective autophagy, Atg11 is outcompeted by its counterpart Atg17, which nucleates autophagosomes independently of cargo.

## Introduction

Macroautophagy, to which we will refer as autophagy in the following, is a pivotal multipurpose recycling system that is essential to maintain cellular homeostasis [1]. During normal cell growth, a membrane structure, termed phagophore, sequentially expands and engulfs cytoplasmic content that is destined for degradation. This cargo is thus selectively sequestered in autophagosomes and delivered to lysosomal compartments [2,3]. The selective recycling of superfluous or damaged cytoplasmic material is essential to maintain cellular homeostasis. Cytotoxic stresses or starvation, however, trigger the formation of autophagosomes that capture cytoplasm nonselectively [4]. Autophagy is thus also essential for cellular survival during periods of adverse environmental conditions. Dysfunctions of the pathway are closely related to the onset of several diseases, such as neurodegeneration or cancer [5,6].

In yeast, autophagosomes form at a distinct location in close proximity to the vacuole, which has been termed phagophore assembly site (PAS [7]). Starvation induces the formation of nonselective autophagosomes upon assembly of a pentameric Atg1 kinase complex (Atg1^PC^). This complex consists of the Atg1 kinase, its regulator Atg13, the scaffolding protein Atg17, as well as Atg31 and Atg29 [8]. Atg17 forms crescent-shaped tail-to-tail dimers [9], which constitutively interact with the two inhibitory subunits Atg31 and Atg29 to form a stable subcomplex (Atg17^TC^) [10]. Upon assembly of Atg1^PC^, Atg17 is activated and tethers Atg9 vesicles to initiate the formation of nonselective autophagosomes [11]. Moreover, Atg29 becomes phosphorylated during starvation, leading to the recruitment of Atg17^TC^ to the PAS in an Atg11-dependent manner [12]. Aspects of this initiation event are conserved from yeast to humans. This includes the Atg1 kinase (Ulk1/2 in humans) and its regulation by Atg13 as well as Atg9 vesicles that supply membranes for the biogenesis of autophagosomes. However, human homologs for Atg17, Atg31, and Atg29 have not yet been described.

Atg11 is an essential scaffolding protein in selective autophagy and connects cargo to the autophagic membrane through its interactions with cargo receptors and with Atg8 at the phagophore [13–15]. Furthermore, the interaction of Atg11 with the Atg1/Atg13 subcomplex stimulates the kinase activity of Atg1 and coordinates tethering of cargo to the phagophore [16]. While Atg17 recruits Atg9 vesicles to the PAS during starvation, Atg11 fulfills this function during selective types of autophagy and organizes the early PAS through its interactions with Atg1, Atg13, cargo receptors, and Atg9 [15–18]. The molecular function of Atg11 during selective autophagy remained, however, unclear.

The presence of the two distinct scaffolding proteins in yeast with Atg11 being involved in selective and Atg17 in nonselective autophagy provides a unique system to study how specificity of autophagy changes in response to environmental cues. Here, we reconstituted the interaction of Atg11 with Atg9-proteoliposomes (Atg9-PLs) from purified components on model membranes in vitro. We found that the autophagy receptor Atg32 activates Atg11 by inducing its dimerization. The Atg11 dimer binds two Atg9 vesicles in vitro, bringing their membranes into close vicinity. Tethering of Atg9 vesicles by Atg11 thus strictly depends on its interaction with cargo. Furthermore, augmented expression of Atg32 in yeast activates Atg11 and stimulates mitophagy while nonselective autophagy is suppressed. Upon starvation, Atg11 is degraded while Atg17 is activated through assembly of the Atg1 kinase complex. This allows Atg17 to sequester Atg9 vesicles and tether them independent of cargo. Taken together, our data provide the molecular basis to understand how selective and nonselective types of autophagy are coordinated at the PAS.

## Results

### Competition of Atg11 and Atg17 at the PAS

**Fig 1:**
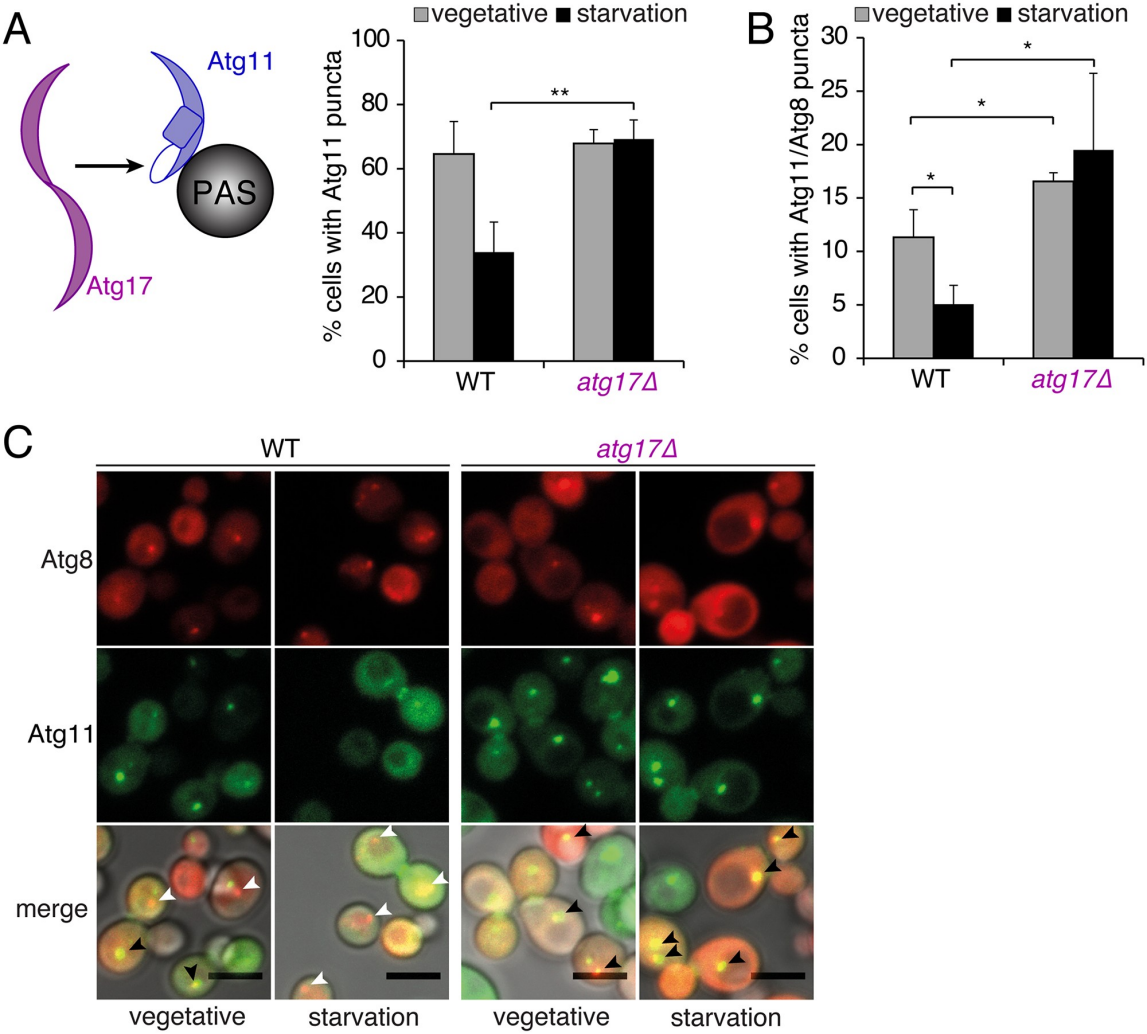
Localization of Atg11 to the PAS is influenced by Atg17. (A) Schematic drawing illustrates the addressed question. Note that the proteins are not drawn to scale and that the cartoon for Atg11 represents a theoretical model since no structural information of Atg11 is available. The percentage of cells with Atg11-GFP puncta was assessed in WT and *atg17*Δ cells during vegetative growth and under starvation. (B) Percentage of cells with GFP-Atg11 puncta that colocalized with mCherry-Atg8 puncta in WT and *atg17Δ* cells during vegetative growth and under starvation. (C) Representative fluorescence images of data shown in (B). Black and white arrowheads indicate positions of Atg8 puncta, which are colocalizing (black) or not colocalizing (white) with Atg11. Scale bars 5 μm. (A and B) Data are presented as mean values ± SD of *n* = 3 independent experiments. Flattened z-stacks of >160 cells per condition were examined using a Fiji script. *P* values were calculated using a two-tailed Student *t* test (*P < 0.05, **P < 0.01). See also S1 Fig. GFP, green fluorescent protein; PAS, phagophore assembly site; WT, wild-type.

The two autophagic scaffolding proteins Atg11 and Atg17 are components of the Atg1 kinase complex [8,16], but Atg11 is essential for selective types of autophagy, whereas Atg17 is required for nonselective autophagy [18–20]. To investigate whether the localization of Atg11 to the PAS is influenced by Atg17, we compared the formation of Atg11 puncta under vegetative and starvation conditions in cells expressing or lacking Atg17. Consistent with the pivotal function of Atg11 in selective autophagy, Atg11 puncta were observed in 65% of cells under vegetative conditions but in only 34% of starved cells (Fig 1A with overexpressed Atg11 for improved visibility and S1A Fig with endogenously expressed Atg11 as control). To confirm that the starvation dependent reduction of Atg11 puncta was caused by the depletion of Atg11 from the PAS, we compared the colocalization of Atg11 and Atg8 in starved and nonstarved cells. We indeed observed a strong and significant decrease in the number of cells with Atg8/Atg11 puncta upon starvation (Fig 1B and 1C, S1B Fig). However, in cells lacking Atg17 no starvation-induced decline of Atg11 puncta was observed (Fig 1A, S1A Fig). Instead, the number of starved *atg17Δ* cells that contained Atg8/Atg11 puncta remained unchanged compared to that in nonstarved *atg17Δ* cells. Thus, starvation has apparently different effects on the number of Atg11/Atg8 puncta in wild-type and *atg17Δ* cells. This results in an overall 4-fold increase in the number of such puncta in starved *atg17Δ* cells compared to starved wildtype cells (Fig 1B and 1C, S1B Fig). Our results thus suggest that in the absence of Atg17, Atg11 remains at the PAS upon starvation.

We next tested whether retention of Atg11 at the PAS in starved *atg17*Δ cells leads to the formation of selective instead of nonselective autophagosomes. We therefore quantified the flux of selective and nonselective cargo in cells expressing or lacking Atg17 by determining the activity of vacuolar Pho8 that was targeted to the cytosol (nonselective) or to mitochondria (selective autophagy). Consistent with previous reports, nonselective autophagy was abolished in cells lacking Atg17, but only moderately decreased in *atg11*Δ cells (S1C Fig). Conversely, no mitophagy was detected in cells lacking Atg11, but significant residual mitophagic activity was observed in *atg17*Δ cells (S1D Fig). These results show that the retention of Atg11 at the PAS upon deletion of Atg17 is sufficient to maintain mitophagy, while nonselective autophagy is severely impaired.

### Scaffold proteins compete for binding of Atg9 in vivo

**Fig 2:**
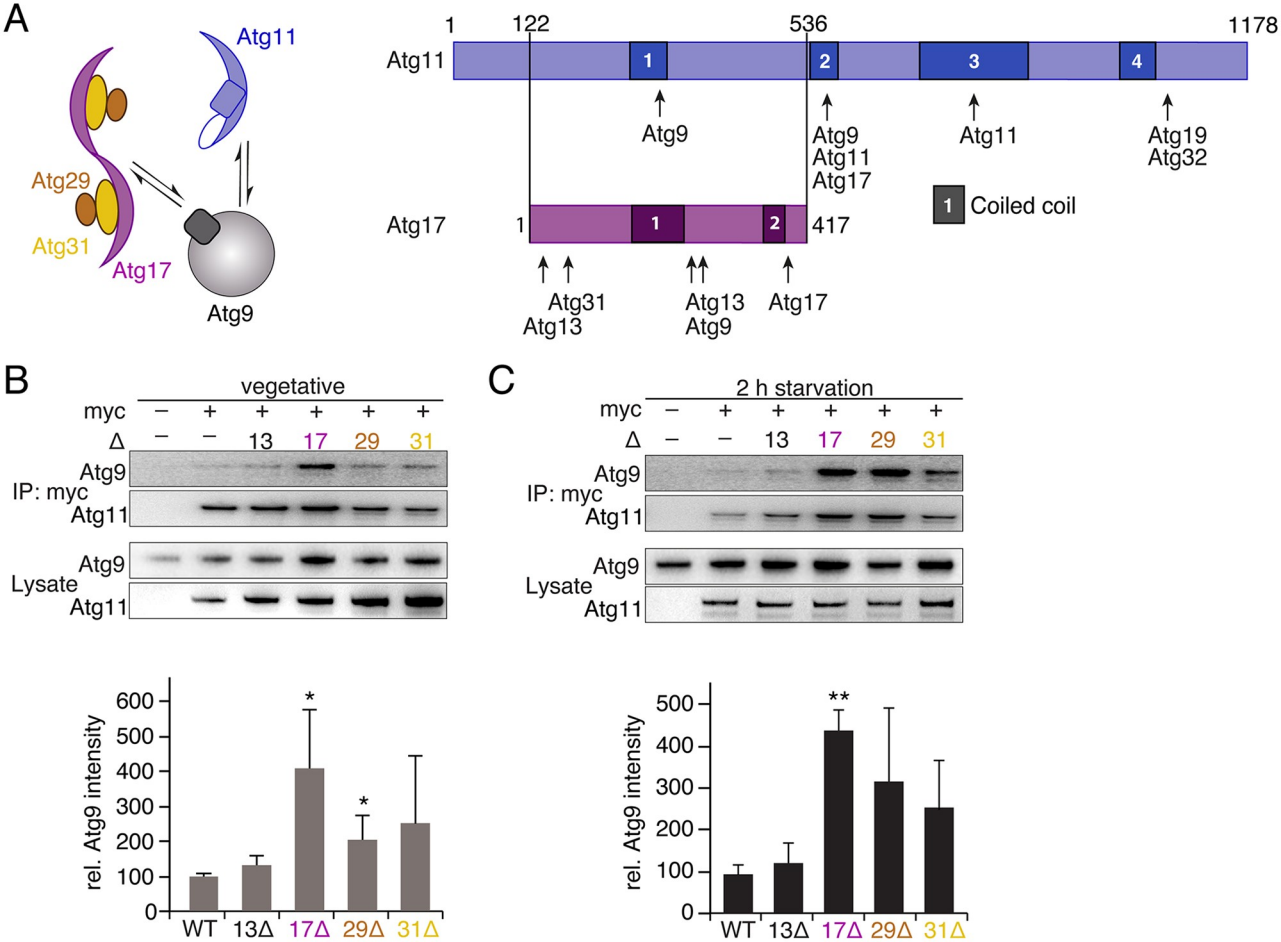
Homologous scaffolds Atg11 and Atg17 compete for binding to Atg9. (A) Schematic drawing illustrates the addressed question. Predicted CC regions of Atg11 and Atg17, and interaction sites with other Atg proteins are indicated. Sequence alignment by ClustalW suggests that Atg17 is homologous to Atg11^122-536^. (B) IP of myc-tagged Atg11 and co-IP of HA-tagged Atg9 from lysates of WT (−), *atg13Δ*, *atg17Δ*, *atg29Δ*, and *atg31Δ* cells (log phase). Lysate of nontagged *atg11* served as control. Atg11 and Atg9 were detected by α-myc and α-HA immunoblots, respectively. The chart shows corresponding quantifications of Atg9 intensities normalized to the WT and corrected for the total amount of Atg11. (C) IP of myc-tagged Atg11 and co-IP of HA-tagged Atg9 as in (B) but from lysates of starved cells. (B and C) Data are presented as mean values ± SD of *n* = 3 independent experiments. *P* values were calculated using a two-tailed Student *t* test (*P < 0.05, **P < 0.01, ***P < 0.001). Uncropped original blots are shown in S5 Fig. CC, coiled–coil; HA, hemagglutinin; IP, immunoprecipitation; WT, wild-type.

A recent reciprocal blast analysis of Atg17 family proteins in different species identified an Atg17-like domain comprising the characteristic signature motif YxxxL/V/IxEV/IxRRR/K [21]. Interestingly, this study reported that Atg11 possesses such an Atg17-like motif and that the N-terminus of Atg11 is remarkably homologous to Atg17 (Fig 2A). Furthermore, Atg17 and Atg11 are known to interact with the transmembrane protein Atg9 [22,23]. We thus asked whether Atg17 and Atg11 compete in addition to their reciprocal recruitment to the PAS also for Atg9 binding. To address this question, we compared binding of Atg11 to Atg9 in wild-type, *atg13*Δ, *atg17*Δ, *atg29*Δ, and *atg31*Δ cells. Interestingly, deletion of Atg17 led to a significantly increased binding of Atg11 to Atg9 under vegetative conditions (Fig 2B). This suggests that the previously observed residual binding of Atg17 to Atg9 in nonstarved cells [11] sequesters a portion of Atg9. More importantly, binding of Atg9 to Atg11 increased by a factor of 4.1 in *atg17*Δ compared to wild-type cells upon starvation. We concluded from these data that Atg17 and Atg11 compete for Atg9 binding and activation of Atg17 upon starvation depletes Atg11 from Atg9. Considering the relatively large standard deviations, a tendency toward increased binding of Atg11 to Atg9 was also observed in cells lacking either Atg29 or Atg31 (Fig 2C), indicating that the integrity of the ternary complex Atg17^TC^ is required to outcompete Atg11 from Atg9.

Interestingly, deletion of Atg13 had no effect on Atg9 binding under either nutrient condition (Fig 2B and 2C). Thus, although the interaction of Atg11 with Atg1-Atg13 is essential for selective autophagy, it is dispensable for binding of Atg11 to Atg9.

### Atg17 and Atg11 directly compete for Atg9 binding in vitro

So far, we could demonstrate that Atg17 negatively regulates the recruitment of Atg11 to the PAS and its association with Atg9 in vivo. Furthermore, previous studies reported that both Atg11 and Atg17 possess Atg9-binding sites [22,24]. Whether these interactions in vivo reflect direct physical binding to Atg9 and whether binding of Atg11 and Atg17 to Atg9 is mutually exclusive remained, however, unknown. In order to address this question, we applied an in vitro reconstitution approach, which was based on purified, recombinant Atg11 and Atg17 as well as synthetic Atg9 vesicles. We have used a similar system to investigate the regulation of the Atg17-containing Atg1 kinase complex and its binding to Atg9 previously [11]. In order to characterize binding of Atg11 to Atg9 vesicles, we here established the recombinant expression and purification of Atg11.

Biophysical characterization of His-tagged Atg11 showed that the protein possesses a molecular weight of 136 kDa (S2A Fig) and eluted at 11.4 ml from a Superose 6 10/300 GL column (GE Healthcare, S2B Fig). Furthermore, sedimentation velocity analysis showed that most of Atg11 sediments with s_20°c,w_ = 6.9 S (S2C Fig) and possesses a Stokes radius of 6 nm as well as a friction factor of 1.77. These findings are consistent with recently reported values for Atg11 [24]. Due to the lack of a crystal structure, the shape of Atg11 is unknown making it difficult to reveal its oligomeric state from velocity sedimentation data. However, we observed that approximately 16% of the total mass of Atg11 possessed a sedimentation coefficient of s_20°c,w_ = 9.4 S and a Stokes radius = 7 nm. The biophysical characteristics of these two species strongly suggest that the majority of Atg11 forms moderately elongated monomers in solution and that a small fraction dimerizes. The elongated nature of monomeric Atg11 is consistent with its predicted secondary structure, which revealed that Atg11 comprises four coiled–coil regions [25].

**Fig 3:**
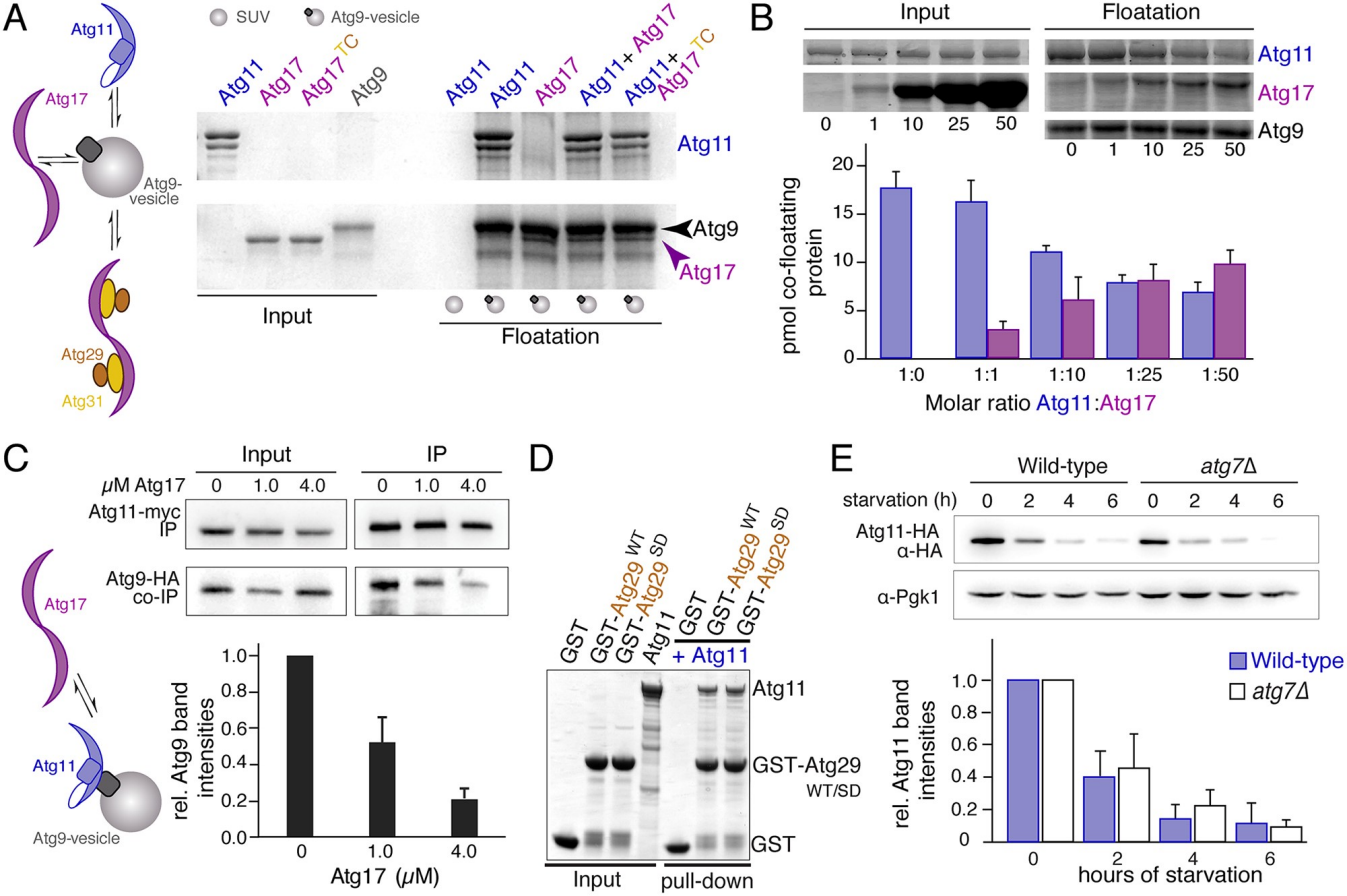
Atg11 and Atg17 compete for Atg9. Schematic drawing illustrates the addressed question but proteins are not drawn to scale. (A) SDS-PAGE gel of input (20% of protein used for cofloatation) and floated fractions from cofloatation experiments of Atg9-PLs with recombinant Atg11, Atg17, and Atg17^TC^. SUVs lacking Atg9^core^ [11] served as control for unspecific membrane binding. (B) SDS-PAGE gel of input (20% of protein used for cofloatation) and floatation fractions from cofloatation experiments of Atg9-PLs with different stoichiometric ratios of recombinant Atg11 and Atg17 as indicated. The chart shows quantifications of relative band intensities as shown in the SDS-PAGE gel above from three independent experiments. Data are shown as mean ± SD. Amounts were calculated using the input band intensities which correspond to known protein quantities as internal reference. (C) Western blots of lysates and samples from immunoprecipitations of nonstarved cells expressing myc-tagged Atg11 and HA-tagged Atg9, using anti-myc and anti-HA antibodies, respectively. Cell lysates were incubated with buffer (0 μM) or purified Atg17 at a final concentration of 1 and 4 μM. Atg11 was immunoprecipitated using anti-myc antibodies bound to Protein A magnetic beads. The chart shows quantification of the relative HA-Atg9 band intensities from three independent experiments, corrected by the amount of immunoprecipitated Atg11-myc and normalized to the control sample (0 μM Atg17). Data are shown as mean ± SD. (D) SDS-PAGE gel of input and pull-down fractions from pull-down assay using 10 μg recombinant GST-tagged WT (Atg29^WT^) or phosphomimetic Atg29 (Atg29^SD^), coupled to Glutathione-resin, with recombinant Atg11. GST served as negative control. Input corresponds to 20% of total protein. Full gel is shown in S5 Fig. (E) Western blots of lysates from wildtype and *atg7* knock-out cells that expressed Atg11-HA using anti-HA antibodies. Pgk1 served as loading control. The chart shows quantifications of blots from three independent experiments, normalized to the amount of Pgk1, relative to the amount in nonstarved cells, which was set to 1. Data are shown as mean ± SD. Uncropped original blots are shown in S5 Fig. GST, glutathione-S-transferase; HA, hemagglutinin; PL, proteoliposome; SUV, small unilamellar vesicle; WT, wild-type.

We next studied the interaction of Atg11, Atg17, and Atg9 in vitro using floatation assays with synthetic Atg9 vesicles. We observed a surprisingly efficient cofloatation of Atg11 with Atg9 vesicles but not with vesicles lacking Atg9. This demonstrates that Atg11 directly binds Atg9 without significant contribution of phospholipids. Moreover, Atg11 was found to bind Atg9 much more efficient than Atg17 or Atg17^TC^ (Fig 3A, S2D Fig). A quantitative analysis using the relative SDS-PAGE band intensities of floating fractions revealed that 18 ± 2 pmol Atg11 were bound to 27 ± 2 pmol Atg9, corresponding to an Atg9 saturation of approximately 70% (Fig 3B). Thus, a stoichiometry of one to one for this interaction can be assumed, which suggests that Atg11 possesses only one Atg9 binding site. We next coincubated stoichiometric amounts of Atg17 and Atg11 with Atg9 vesicles. Consistent with a high avidity of Atg11 to Atg9-binding, we recovered 16 ± 2 pmol Atg11 but only 3 ± 1 pmol Atg17 from floating fractions. The saturation of Atg9 vesicles remained constant (approximately 70%), demonstrating that Atg11 outcompetes Atg17 in vitro (Fig 3B). In a next step, we gradually increased the amount of Atg17 in floatations while the amount of Atg11 was kept constant. We observed that a 25-fold molar excess of Atg17 was required to recover similar amounts of both proteins from floatation fractions (Fig 3B). This suggests that even in context of the fully assembled Atg1-kinase complex, a stoichiometric excess of Atg17 would be required to outcompete Atg11 from the PAS during starvation. We next investigated, whether a stoichiometric excess of Atg17 is also required to outcompete Atg11 from Atg9 in cell lysates. We therefore added increasing amounts of Atg17 to lysates of nonstarved yeast cells and coimmunoprecipitated HA-tagged Atg9 with myc-tagged Atg11. We observed that at an Atg17 concentration of 1μM, approximately 50% of Atg11 was displaced from Atg9 (Fig 3C). Based on the copy number of Atg11 per cell (750 molecules [26]) and the average volume of a yeast cell (approximately 100 fl [27]), we estimated that the Atg11 concentration in our lysates (diluted total cell lysates) is approximately 40 nM. Taking these numbers into account, we concluded that a 25-fold molar excess of Atg17 is sufficient to achieve equal binding of Atg11 and Atg17 to Atg9 in cell lysates, which agrees well with the value obtained from floatation experiments in vitro. When we further increased the Atg17 concentration to 4 μM, approximately 80% of the Atg9-binding sites were occupied by Atg17, demonstrating that Atg17 displaces Atg11 from Atg9 vesicles in a concentration-dependent manner. Taken together, our experiments show that Atg17 is a competitive inhibitor of Atg11 and that both scaffolds use at least partially overlapping binding sites. Furthermore, an approximately 25-fold stoichiometric excess of Atg17 was outcompeting Atg11 from Atg9 in vitro and in cell lysates. The identical inhibitory capacity of recombinant Atg17 in vitro and in cell lysates also demonstrates that the affinity of purified Atg11 to reconstituted Atg9 in vitro is similar to that of endogenous Atg11 to Atg9 in cell lysates.

Quantitative mass spectrometry studies in yeast reported similar copy-numbers of Atg11 and Atg17 in nonstarved cells [28]. This implies that Atg17 cannot outcompete Atg11 under these conditions. We reasoned that additional regulatory mechanisms must exist to ensure an efficient sequestration of Atg9 by Atg17 upon starvation. A previous study reported that phosphorylation of Atg29 regulates the recruitment of Atg17 to the PAS in an Atg11-dependent manner [12]. We thus tested whether purified Atg29 or its corresponding phosphomimetic variant Atg29^SD^ (Atg29^S197D, S199D, S201D^, [12]) interacts with Atg11 in vitro by coprecipitating purified Atg11 using GST-Atg29^WT/SD^ or GST-Atg31-Atg29^WT/SD^ as baits. Interestingly, Atg11 strongly interacted with both Atg29 variants independently of Atg31 (Fig 3D, S2E Fig). This suggests that although the recruitment of Atg17 to the PAS might be promoted by a physical interaction of Atg29 and Atg11, this interaction is apparently not regulated in a starvation-dependent manner.

Another evidence for a potential regulatory mechanism came from our immunoprecipitation (IP) experiments, in which we observed significantly reduced amounts of Atg11 in lysate fractions from starved cells compared to that of nonstarved controls. We thus systematically analyzed expression levels of Atg17 and Atg11 in nonstarved cells and during starvation. Consistent with previous studies [29], the amount of Atg17 remained constant upon starvation (S2F Fig). By contrast, the level of Atg11 declined in a time-dependent manner after autophagy induction (Fig 3E). Since starvation induces the formation of nonselective autophagosomes, the detected decline in Atg11 could be caused by a passive autophagy-dependent transport of Atg11 together with bulk cytoplasm to the vacuole. We thus analyzed Atg11 levels in autophagy-deficient *atg7Δ* cells. Interestingly, a similar reduction of Atg11 levels was observed, demonstrating that the degradation of Atg11 upon starvation is autophagy-independent (Fig 3E). To test whether Atg11 is degraded in the vacuole, we compared Atg11 levels in lysates from cells in which the vacuolar enzyme pep4 was deleted. We observed a similar decline in Atg11 levels upon starvation as in wild-type cells, confirming that the degradation of Atg11 is independent of vacuolar targeting pathways (S2G Fig).

An alternative mechanism by which proteins can be degraded in a regulated manner involves the ubiquitin (Ub)-proteasome system. We reasoned that if this system is involved in Atg11 degradation, a significant increase in ubiquitinated Atg11 should be observable upon starvation. To test this hypothesis, we first purified ubiquitinated proteins from lysates of Atg11-myc–expressing cells followed by detection of Atg11 by western blot analysis using anti-myc antibodies. No Atg11 was detected in IP fractions from lysates of cells expressing untagged Atg11 or if uncharged beads were used, confirming the specificity of our assay. Interestingly, lysates from starved cells contained 2.6 times more ubiquitinated Atg11-myc compared to that from nonstarved cells, although the total amount of Atg11 strongly declined upon starvation (S2H Fig). To finally prove that Atg11 is indeed degraded by the proteasome upon starvation, we compared Atg11 levels in cells that have been treated with the proteasomal inhibitor MG-132 to those in wild-type cells. We found that in the presence of MG-132, Atg11 levels remained constant in starved and nonstarved cells (S2I Fig). Our data thus show that upon induction of autophagy by starvation, Atg11 becomes ubiquitinated and is degraded by the proteasome.

Interestingly, the amount of Atg11 in cell lysates declined by a factor of 10 to 100 upon starvation while that of Atg17 remained constant (Fig 3E). Given that the concentrations of Atg17 and Atg11 are similar in nonstarved cells [26], the selective degradation of Atg11 upon starvation leads to a molar excess of Atg17. Our data thus provide a plausible mechanism by which starvation induces the sequestration of Atg9 vesicles by Atg17. Moreover, we could show that the proteasomal degradation of Atg11 upon starvation is a key element for switching from selective to nonselective autophagy.

### Atg11 contains an Atg17-like tethering domain

**Fig 4:**
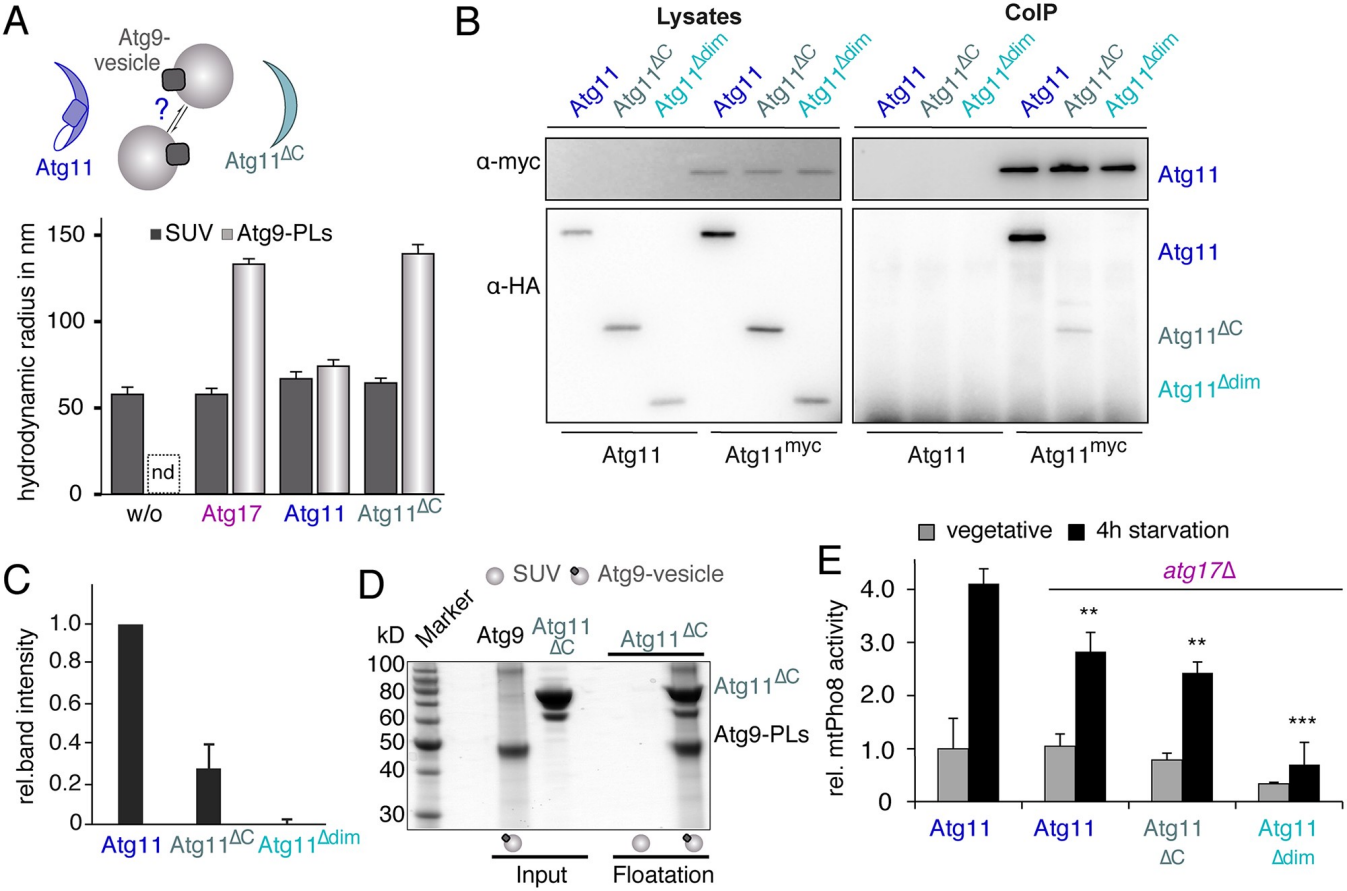
The N-terminus of Atg11 tethers Atg9-PLs. (A) The R_H_ of SUVs (dark grey bars) and Atg9-PLs (light grey bars) was determined using DLS in the absence and presence of Atg17, Atg11, and Atg11^ΔC^. Atg9-PLs are polydisperse in the absence of interaction partners, preventing the reliable determination of their size by light scattering. (B) The dimerization site in Atg11 was identified by IP myc-tagged full length Atg11 and co-IP of HA-tagged Atg11-variants as indicated from lysates of log-phase growing cells. Lysates of untagged Atg11 served as control. Atg11 and its fragments were detected by α-myc and α-HA immunoblots, respectively. (C) The chart shows quantification of band intensities of anti-HA blots shown in B. Values show the band intensity of co-IP Atg11 variants as a fraction of total protein levels in lysates, normalized by the amount of immunoprecipitated Atg11-myc. Values of full-length Atg11-HA were set to 1. Data are presented as mean values ± SD from three independent experiments. (D) SDS-PAGE gel with input (100% of protein used for cofloatation) and floated fractions from cofloatation assay of Atg9-PLs with Atg11^ΔC^. SUVs lacking Atg9^core^ serve as control for unspecific membrane binding. (E) Mitochondrial Pho8ΔN60 (mtPho8) assay of log-phase growing and starved *atg17Δ* cells expressing Atg11^WT^, Atg11^ΔC^, or Atg11^Δdim^. Pho8-activity was corrected for total protein amount of each sample and normalized to the signal of log-phase WT cells (set to 1). Data are presented as mean values ± SD of *n* = 5 (A) and *n* = 3 (E) independent experiments. *P* values were calculated using a two-tailed Student *t* test (*P < 0.05, **P < 0.01, ***P < 0.001). DLS, dynamic light scattering; HA, hemagglutinin; IP, immunoprecipitating; nd, not determined; PL, proteoliposome; R_H_, hydrodynamic radius; SUV, small unilamellar vesicle; WT, wild-type.

We previously demonstrated that Atg17 tethers Atg9 vesicles in vitro and that the tethering function of Atg17 is critical for the nucleation of autophagosomes under starvation [11]. Here, we showed that Atg11 and Atg17 compete for Atg9 binding and for their localization to the PAS, suggesting that both proteins execute similar functions. We thus tested whether Atg11 does not only bind Atg9 vesicles in vitro but also tethers such vesicles. We first incubated Atg17 with Atg9 vesicles or with small unilamellar vesicles (SUVs, lacking Atg9) and measured their hydrodynamic radius by dynamic light scattering. Consistent with our previous report, we observed a 2-fold increase in the hydrodynamic radius of Atg9 vesicles compared to control SUVs, showing that Atg17 tethers Atg9 vesicles. By contrast, similar hydrodynamic radii were observed for SUVs and Atg9 vesicles in the presence of Atg11 (Fig 4A). This demonstrates that although Atg11 is able to bind Atg9, it cannot tether Atg9 vesicles. We reasoned that the monomeric state of Atg11 (S2C Fig) combined with the presence of only one Atg9 binding site per Atg11 molecule (Fig 3B) prevents tethering. This suggests that Atg11 would need to dimerize in order to tether Atg9 vesicles.

Interestingly, previous studies showed that both Atg11 and Atg17 form functionally relevant oligomers in vivo [9,15]. The corresponding self-interaction sites in Atg11 were mapped to coiled–coil regions two and three [15]. However, the N-terminus of Atg11 contains an Atg17 homologous domain (residues 122–536) that also encompasses a region that mediates Atg17 dimerization (Fig 2A). We thus asked whether the N-terminus of Atg11 (Atg11^ΔC^, residues 1–667, S3A Fig) possesses an additional self-interacting motif. We therefore coimmunoprecipitated HA-tagged Atg11^ΔC^ using myc-tagged Atg11 as bait. We observed that Atg11^ΔC^ indeed interacts with Atg11, although its efficiency is decreased by 70% compared to that of full-length Atg11 (Fig 4B and 4C). If we, however, truncated Atg11^ΔC^ further to exclude the Atg17-like dimerization domain (Atg11^Δdim^, S3A Fig), binding to full-length Atg11 was entirely abrogated (Fig 4B and 4C). Our results thus show that the N-terminal domain of Atg11 contains an Atg17-like self-interaction motif, which promotes the formation of Atg11 oligomers in vivo.

In order to determine whether the observed self-interaction of Atg11^ΔC^ in vivo corresponds to the formation of defined Atg11^ΔC^ oligomers, we determined the oligomeric state of purified Atg11^ΔC^ by size exclusion chromatography and analytical ultracentrifugation in vitro. We found that although Atg11^ΔC^ is with a molecular weight of 78.2 kDa much smaller than Atg11, it elutes earlier from a Superose 6 column, indicating that Atg11^ΔC^ forms dimers. Moreover, we observed a concentration-dependent shift of the sedimentation coefficient of Atg11^ΔC^ from s_20°c,w_ = 4.4 S at low concentration to s_20°c,w_ = 4.7 S at high concentration. By contrast, identical, concentration-independent sedimentation coefficients were observed for full-length Atg11 (s_20°c,w_ = 5.4 S, S3B Fig). This shows that purified Atg11^ΔC^ indeed forms dimers in solution while Atg11 is mostly monomeric. Further, the constitutive assembly of Atg11^ΔC^ dimers in vitro suggests that the strongly reduced formation of Atg11-Atg11^ΔC^ heterodimers in vivo is caused by a highly efficient formation of Atg11^ΔC^ homodimers that sequester most of Atg11^ΔC^ (Fig 4B and 4C).

We next tested whether the integrity of the Atg17-like domain of Atg11 is required and sufficient for an interaction with Atg9 by coimmunoprecipitating Hemagglutinin (HA)-tagged Atg9 using green fluorescent protein (GFP)-tagged Atg11^ΔC^ and Atg11^Δdim^ as baits. Interestingly, binding of both Atg11 fragments to Atg9 was much weaker compared to that of Atg11 in growing cells but was almost fully restored upon starvation (S3C Fig). This surprising difference might indicate that the C-terminal domain of Atg11 possesses an important regulatory function. In order to establish that the detected interaction of Atg11^ΔC^ with Atg9 in vivo corresponds to a physical interaction of both proteins in vitro, we performed cofloatation experiments with purified components. Atg11^ΔC^ was indeed cofloating with Atg9-PLs (Fig 4D).

Our observation that Atg11^ΔC^ forms dimers and directly interacts with Atg9 implies that Atg11^ΔC^ tethers Atg9 vesicles in vitro. We thus compared the hydrodynamic radii of Atg9 and control vesicles in the presence of Atg11^ΔC^. Interestingly, the radius of Atg9 vesicles (R_H_ = 140 ± 5 nm) increased by a factor of two compared to control vesicles (68 ± 2 nm; Fig 4A), demonstrating that dimerization of Atg11^ΔC^ is sufficient to tether Atg9 vesicles in vitro.

The constitutive dimerization of Atg11^ΔC^ and its propensity to tether Atg9 vesicles in vitro implies that Atg11^ΔC^ constantly promotes selective autophagy in vivo. However, previous studies demonstrated that deletion of the C-terminal domain of Atg11 abrogates selective autophagy [15,30]. This inhibition can either be caused by a functional inactivation of Atg11^ΔC^ or by the lack of critical cargo-binding sites that are located in the C-terminal domain. In order to distinguish between both possibilities, we tested whether the formation of hetero-dimers between full-length Atg11 and Atg11^ΔC^ is sufficient to promote selective autophagy using mito-Pho8 assays (Fig 4E). The rationale behind this approach was that Atg11 provides cargo-binding sites in Atg11- Atg11^ΔC^ hetero-dimers, while monomeric Atg11^Δdim^ lacks such binding sites but retains its capacity to bind Atg9. In order to untangle Atg11 mediated mitophagy from nonselective degradation of mitochondria in an Atg17-dependent manner, we performed these experiments in *atg17*Δ cells. We found that in cells that coexpressed Atg11 and Atg11^ΔC^ mitophagy was only slightly reduced (82 ± 2%) compared to cells expressing full-length Atg11. We concluded that the formation of Atg11-Atg11^ΔC^ hetero-dimers is sufficient to promote selective autophagy (Fig 4E). By contrast, mitophagy was strongly impaired upon coexpression of Atg11 and Atg11^Δdim^ (44 ± 1% of control), suggesting that monomeric Atg11^Δdim^ is a competitive inhibitor of Atg11.

Previous studies demonstrated that starvation strongly enhances mitophagy in an Atg11 (selective) and Atg17 (nonselective) dependent manner [31]. Consistently, we observed that starvation led to an increase of mitophagy by a factor of four in cells that expressed Atg11 and Atg17. Moreover, mitophagy was reduced by approximately 30% in cells that coexpressed Atg11^ΔC^, full-length Atg11, and Atg17, which compares to values observed in *atg17Δ* cells. This indicates that expression of Atg11^ΔC^ leads to a moderate reduction in mitophagy independently of Atg17. However, expression of Atg11^Δdim^ led to a weaker inhibition of mitophagy in Atg17 expressing cells (approximately 70% of Atg11) compared to that in *atg17Δ* cells (approximately 20% of Atg11), suggesting that the inhibitory effect of Atg11^Δdim^ is compensated by Atg17. Taken together, our data show that the Atg17-dependent (nonselective) degradation of mitochondria dominates Atg11-dependent (selective) mitophagy during starvation (S3D Fig).

The strikingly similar properties of Atg17 and Atg11^ΔC^ in vitro suggest that Atg11^ΔC^ might be able to functionally complement a deletion of Atg17 in starved cells. We therefore quantified nonselective autophagy in nonstarved and starved *atg17Δ* cells that coexpressed Atg11 and either Atg11^ΔC^ or Atg11^Δdim^ (S3E Fig). However, expression of Atg11^ΔC^ did not rescue the autophagic defect in *atg17Δ* cells. Thus, although Atg11^ΔC^ and Atg17 share similar properties in terms of their dimeric state and their binding to and tethering of Atg9 vesicles, Atg11^ΔC^ cannot replace Atg17 at the PAS.

### The mitochondrial cargo receptor Atg32 activates Atg11

We demonstrated here that the N-terminal domain of Atg11 shares striking functional similarities to Atg17, while its C-terminal domain plays an important regulatory role and inhibits Atg11. Interestingly, the C-terminus of Atg11 contains also the binding site for the mitochondrial receptor Atg32 [30], which is anchored within the outer membrane of mitochondria by a C-terminal transmembrane helix. The degradation of mitochondria through selective autophagy is initiated upon phosphorylation of Atg32 at serine 114 [30]. To test how binding of Atg32 influences the activity of Atg11 in vitro, we recombinantly expressed and purified the N-terminal cytoplasmic domain of Atg32 (Atg32^1-376^) as well as the corresponding phosphomimetic mutant S114E (Atg32^SE^ in the following). We first reconstituted the recruitment of Atg11 and Atg32 to the phagophore in vitro. In order to mimic the phagophore membrane, we conjugated Atg8 to giant unilamellar vesicles (GUVs) using the purified Ub-like conjugation system [32]. Atg32 possesses an Atg8-interacting motif (AIM), which crosslinks mitochondria and phagophore during mitophagy [33]. Purified Atg32^SE^ was indeed efficiently recruited to Atg8-GUVs, whereas a corresponding Atg32^1-376^ variant in which the AIM motif was mutated (Atg32^WG^) [33] did not bind such GUVs (S4A Fig). Moreover, a recent study reported that the Arabidopsis homolog of Atg11 (AtATG11) directly interacts with AtATG8 [21]. An interaction of the corresponding yeast homologs has, however, not been reported. To test whether Atg11 directly binds Atg8, we incubated Atg8-GUVs and GUVs lacking Atg8 with fluorescent-labeled Atg11. Remarkably, Atg11 colocalized with Atg8-positive membranes but not with membranes lacking Atg8, demonstrating that Atg11 directly binds Atg8 in vitro (S4B Fig).

**Fig 5:**
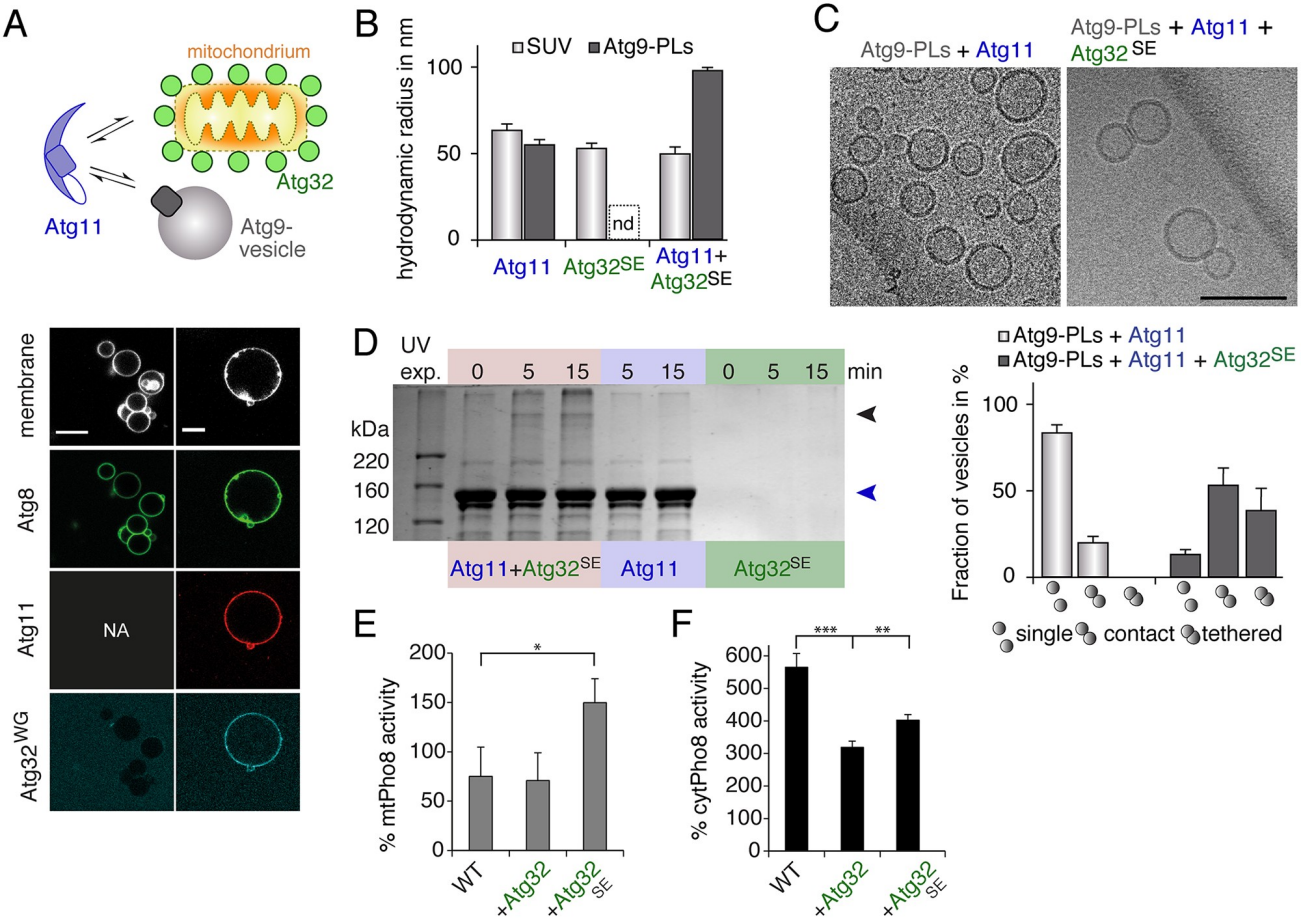
The cargo receptor Atg32 activates Atg11. (A) Atg8^Atto488^, enzymatically coupled to Atto633-labeled GUVs, fails to recruit PacificBlue-labeled Atg32, in which the AIM is mutated (Atg32^WG^). Recombinant Atg11^Atto565^ recruits Atg32^WG^ to Atg8-decorated GUVs. Scale bar 10 μm. (B) The R_H_ of SUVs (dark grey bars) and Atg9-PLs (light grey bars) as obtained in DLS experiments in the presence of either Atg11 or Atg32^SE^ or of both. R_H_ of polydisperse samples is not determinable (nd). (C) Cryo-EM of mixtures of Atg9 vesicles with Atg11 or with Atg11 and Atg32^SE^ as indicated. The chart shows the number of single, contact, and tethered vesicles as fraction of all vesicles of the indicated sample. At least 40 vesicles were analyzed in each of three pools. One pool represents data from different EM grids of identical samples. Single vesicles do not contact any other vesicle; contact vesicles are in close proximity to other vesicles with an apparent contact between membranes. Tethered vesicles have a flattened and extended contact area as shown in the corresponding image. Scale bar 100 nm. (D) SDS-PAGE of samples from cross-linking experiment using the UV-activatable reagent Sulfo-LC-SDA with a spacer arm of 12.5 Å. Sulfo-LC-SDA was conjugated to primary amines in Atg32^SE^. Atg11, Sulfo-LC-Atg32^SE^ or mixtures of both were exposed to 365 nm UV light for indicated times. The blue arrowhead indicates the band corresponding to Atg11 and the black arrowhead that of the crosslinked species with an apparent molecular weight of approximately 350 kDa. (E and F) Mitochondrial Pho8ΔN60 assay (E, mtPho8) of log-phase growing and cytoplasmic Pho8ΔN60 assay (F, cytPho8) of starved WT cells and cells overexpressing Atg32 or Atg32^SE^ lacking its transmembrane domain. Pho8 activity was corrected for total protein amount of each sample and normalized to the signal of WT cells (100%). (B) Data are presented as mean values ± SD of *n* = 5 measurements. (E and F) Mean values ± SD of *n* = 3 independent experiments are shown. *P* values were calculated using a two-tailed Student *t* test (*P < 0.05, **P < 0.01). See also S4 Fig. AIM, Atg8-interacting motif; DLS, dynamic light scattering; EM, electron microscopy; GUV, giant unilamellar vesicle; PL, proteoliposome; R_H_, hydrodynamic radius; SUV, small unilamellar vesicle; WT, wild-type.

We next tested whether Atg32 physically interacts with Atg11. Classical approaches to determine the formation of complexes in solution, including size-exclusion chromatography or thermophoresis, failed to confirm that both proteins directly interact. Since a previous study reported that the interaction of cargo receptors with Atg8 depends on a high local concentration of Atg8 in vitro [34], we tested whether a similar concentration-dependent interaction exists for Atg11 and Atg32. We therefore enriched fluorescent-labeled Atg11 on Atg8-GUVs, followed by coincubation with fluorescent-labeled Atg32. To exclude a direct interaction of Atg32 and Atg8, we used the Atg32^SE,WG^ mutant for these experiments. We found that Atg32^SE,WG^ indeed colocalized with Atg8-GUVs in an Atg11-dependent manner (S4C Fig). Interestingly, a similar dependency on Atg11 was observed for the recruitment of an Atg32^WG^ variant that contained only the mutation of the AIM motif but not the phosphomimetic mutation (Fig 5A). This observation indicates that although receptor phosphorylation is required for mitophagy in vivo, it is dispensable for an interaction of Atg32 with Atg11 in vitro.

Our study revealed that Atg11 is autoinhibited by its C-terminal domain. Moreover, we found that Atg32 physically interacts with Atg11 in vitro. We thus reasoned that Atg32 might release the inhibition of Atg11. We therefore tested whether a mixture of Atg32^SE^ and Atg11 is able to tether Atg9 vesicles by dynamic light scattering. According to our previous results, Atg11 alone did not tether Atg9 vesicles in vitro (Fig 5B). Moreover, SUVs lacking Atg9 possessed comparable particle sizes in the presence of Atg32^SE^ (R_H_ = 53 ± 1 nm) or mixtures of Atg32^SE^ and Atg11 (R_H_ = 50 ± 1 nm). By contrast, coincubating Atg9 vesicles with Atg32^SE^ and Atg11 increased the R_H_ by a factor of two (R_H_ = 94 ± 1 nm, Fig 5B). The increased size of Atg9 vesicles is already a strong indicator for vesicle tethering. To confirm that Atg11 indeed tethers Atg9 vesicles in the presence of Atg32^SE^, we imaged vesicle suspensions by cryo-electron microscopy (cryo-EM). Atg9 vesicles that were incubated with only Atg11 displayed diameters of 25–50 nm and 81 ± 4% of vesicles were found to be dispersed on EM grids. A small fraction of 20 ± 4% of vesicles were in contact with the membrane of another vesicle without forming apparently tight membrane contacts (Fig 5C). However, if we coincubated Atg11 and Atg32^SE^ with Atg9 vesicles, the fraction of nontethered vesicles declined strongly, whereas 51 ± 10% of vesicles were in direct contact to another vesicle. Moreover, 37 ± 12% of vesicles formed tight and intimate membrane contact sites as shown in Fig 5D. Interestingly, Atg17 was previously observed to tether Atg9 vesicles in a similar fashion [11], which underscores our data that both proteins exert similar if not identical functions. In contrast to Atg17, which is active by itself but inhibited if in complex with Atg31-Atg29, we found that tethering of Atg9 vesicles by Atg11 strictly depends on Atg32.

The monomeric nature of Atg11 combined with the presence of only one Atg9-binding site requires Atg11 to dimerize in order to tether Atg9 vesicles. We thus tested whether Atg32 induces the dimerization of Atg11. We therefore conjugated the UV-crosslinker Sulfo-LC-SDA to Atg32^SE^, added Atg11, and exposed the mixture to UV light. Analyzing the products of the crosslinking reaction by SDS-PAGE revealed the appearance of a high molecular weight band in samples that contained Atg11 and Sulfo-LC-Atg32^SE^, indicating that Atg32 induces the formation of Atg11 oligomers. The intensity of this band correlated with the time of UV radiation and was not detected in samples that were not exposed to UV light (Fig 5D). Using the relative migration distances of the marker proteins as standard, the molecular weight of this band was estimated to be approximately 350 kDa. This suggests that the band corresponds well to an Atg11 dimer (265 kDa) that was cross-linked by one or two Atg32 molecules (42 kDa). Moreover, this band was not observed if we exposed only Atg11 or only Sulfo-LC-Atg32^SE^ to UV light, demonstrating that crosslinking of Atg11 was induced in an Atg32-dependent manner (Fig 5D).

Our in vitro reconstitution experiments revealed that Atg32 activates Atg11 by inducing its dimerization. To test, whether Atg32 activates Atg11 also in vivo, we overexpressed Atg32^1-376^ or Atg32^1-376,SE^ in yeast cells and quantified mitophagic and autophagic fluxes. Consistent with an Atg32-dependent stimulation, mitophagy was significantly induced upon overexpression of phosphomimetic Atg32^1-376,SE^ but not by overexpressing Atg32^1-376^ (Fig 5E). Thus, despite the fact that phosphorylation of Atg32 is dispensable for its interaction with Atg11 in vitro, it is essential to activate mitophagy in vivo. Interestingly, we observed that nonselective autophagy was significantly reduced independently of the expressed Atg32 variant (Fig 5F). Our data thus demonstrate that overexpressed Atg32 activates mitophagy while it suppresses nonselective autophagy.

## Discussion

Cellular homeostasis depends on the precise and efficient sequestration of damaged or superfluous cytoplasmic components by autophagosomes. As a consequence, recognition of such material and initiation of selective autophagy need to be coordinated in time and space to avoid accumulation of toxic components, such as damaged mitochondria. How this coordination takes place remained, however, an open question. A key regulator for selective autophagy is Atg11, which recruits Atg9 vesicles to the PAS to initiate phagophore formation. Furthermore, Atg11 recognizes cargo by interacting with cargo receptors, such as the mitochondrial receptor Atg32. Here, we investigated how the two functions of Atg11, i.e., cargo recognition and Atg9 recruitment, are coordinated at a molecular level. The most significant result of our study is the observation that Atg11 tethers Atg9 vesicles in a cargo-dependent fashion.

We previously reported that Atg17 tethers Atg9 vesicles independently of cargo, suggesting that Atg17 and Atg11 exert redundant functions that are apparently differently regulated. We thus characterized binding of Atg11 to Atg9 in more detail by applying an in vitro reconstitution approach based on synthetic Atg9 vesicles and purified recombinant proteins. We revealed that Atg11 possesses a previously uncharacterized Atg9 binding site within the N-terminal Atg17-like domain. Furthermore, we found that Atg11 bound Atg9 much more efficiently than Atg17. If stoichiometric amounts of both proteins were coincubated with Atg9 vesicles, Atg11 was sequestering approximately 60% but Atg17 only 10% of Atg9-binding sites. We concluded that Atg11 is able to outcompete Atg17 from Atg9 at least in vitro. This agrees well with the observation that Atg11 localizes to the PAS and efficiently interacts with Atg9 in nonstarved cells.

Atg17 forms constitutive dimers that tether Atg9 vesicles independent of other cofactors but this activity is inhibited by the regulatory Atg31-Atg29 subcomplex [10,35]. Starvation induces the formation of the pentameric Atg1 kinase complex in which the full capacity of Atg17 to bind and tether Atg9 vesicles is restored [8,11]. Our observation that Atg11 binds Atg9 much stronger than active Atg17 suggests that Atg17 is even as part of the Atg1-kinase complex not able to deplete Atg11 from Atg9 vesicles. However, starvation induces the displacement of Atg11 from the PAS in an Atg17-dependent manner, which coincides with an Atg17-dependent decrease in the interaction of Atg11 and Atg9. This imposes the question how Atg17 outcompetes Atg11 upon starvation.

Interestingly, we found that Atg11 levels strongly decline during starvation whereas those of Atg17 remain constant. Furthermore, we observed that Atg11 degradation occurs in an autophagy-independent fashion and does not depend on vacuolar hydrolases. These results suggest that Atg11 is degraded by the proteasome. Consistent with this hypothesis, we found that the amount of ubiquitinated Atg11 increases upon starvation although the total level of Atg11 was decreased. Moreover, Atg11 was not degraded in cells that were treated with the proteasome inhibitor MG-132. We concluded from these data that starvation triggers the degradation of Atg11 by the Ub–proteasome system.

A global analysis of protein levels in nonstarved yeast cells reported similar copy numbers for Atg17 and Atg11 per cell [28], implying that the selective degradation of Atg11 upon starvation leads to a molar excess of Atg17. We indeed observed that Atg11 levels declined by a factor of 10 to 100 upon starvation, while those of Atg17 remained constant. Moreover, we found that a 25-fold stoichiometric excess of Atg17 is sufficient to permit equal binding of Atg17 and Atg11 to Atg9 vesicles in vivo and in vitro. We furthermore showed that a larger stoichiometric excess of Atg17 outcompetes Atg11 by sequestering Atg9 vesicles in vivo and in vitro. Our data thus imply that the regulated degradation of Atg11 is a key step during the transition from selective to nonselective autophagy upon starvation.

Furthermore, we identified a dimerization motif in the N-terminal domain of Atg11 which is similar to that found in Atg17. However, full-length Atg11 was monomeric in solution and did not tether Atg9 vesicles, whereas its purified Atg17-like domain formed dimers and tethered Atg9 vesicles. We concluded from this observation that the C-terminal domain prevents Atg11 dimerization and thus tethering of Atg9 vesicles. Importantly, we could show that even monomeric Atg11 outcompetes Atg17 in vivo and in vitro. This suggests that the sequestration of Atg9 vesicles by Atg11 is an important element to prevent the formation of nonselective autophagosomes.

But how is the apparent autoinhibition of Atg11 released? Apart from its inhibitory function, the C-terminal domain of Atg11 harbors many known binding sites of autophagy receptors [15,30]. We thus investigated whether the mitochondrial receptor Atg32 regulates the activity of Atg11 and found that binding of Atg32 to Atg11 induces its dimerization and facilitates tethering of Atg9 vesicles in vitro. When we overexpressed Atg32^1-376,SE^ in yeast, selective autophagy was greatly enhanced while starvation induced nonselective autophagy was strongly suppressed. However, if we expressed an Atg11 variant that lacks the dimerization domain (Atg11^Δdim^), mitophagy was greatly reduced. We concluded that dimerization of Atg11 is essential to tether Atg9 vesicles in vitro and to promote selective autophagy in vivo.

Taken together, we propose a model in which in nonstarved cells, Atg11 efficiently sequesters Atg9 vesicles and outcompetes Atg17 to prevent the formation of nonselective autophagosomes. Furthermore, Atg17 is inhibited by forming the constitutive Atg17-Atg31-Atg29 complex. Autophagic cargo, for example damaged mitochondria, activate Atg11 through a direct interaction of autophagy receptors such as Atg32 with the C-terminal domain of Atg11. This allows Atg11 to tether Atg9 vesicles thereby nucleating phagophores in close vicinity to cargo. Thus, Atg11 appears to spatiotemporally coordinate selective autophagy by coupling the presence of autophagic cargo to the initiation of selective autophagy. Upon starvation, Atg11 is degraded and the formation of the Atg1 kinase complex leads to an activation of Atg17. Jointly, these two events permit Atg17 to sequester and tether Atg9 vesicles independently of cargo to induce the formation of nonselective autophagosomes.

## Materials and methods

Supporting methods and corresponding references can be found in S1 Text. All data presented as charts are summarized in S1 Data including measured values, calculated mean values and standard deviations, as well as statistical significance calculations.

### Reagents

The following synthetic lipids were purchased from Avanti Polar Lipids: 1-palmitoyl-2-oleoyl-sn-glycerol-3-phosphocholine (POPC), 1-palmitoyl-2-oleoyl-sn-glycerol-3-phosphoethanolamine (POPE), 1-palmitoyl-2-oleoyl-sn-glycerol-3-phosphoserine (POPS), 1-palmitoyl-2-oleoyl-sn-glycerol-3-phosphoinositol (POPI), cholesterol, and the detergent n-Dodecyl-N,N-Dimethylamine-N-Oxide (LDAO) was from Anatrace. The following antibodies were used in this study: αHA (Santa Cruz, sc-7392, 1:200 dilution,), αMyc (Santa Cruz, sc-789 and sc-40, 1:1,000 dilution), αUb (mono- and poly-ubiquitinylated proteins, Enzo, BML-PW8810, 1:500 dilution), αPgk1 (Invitrogen, 459250, 1:1,000 dilution), and αGFP (Roche, 11814460001, 1:1000 dilution).

### Yeast strains and plasmids

Derivatives of *Saccharomyces cerevisiae* BY4741 and BY4742 (Euroscarf) used for each experiment of this study are listed with genotypes in S1 Table. Plasmids used in this study are specified in S2 Table. The combination of plasmids and strains used for experiments are summarized in S3 Table. Genomic gene deletion and tagging was performed as described previously [36].

### Pull-down and IP experiments

Yeast cells corresponding to 50–100 OD_600_ (optical density at 600 nm) were collected by centrifugation (3 min, 1,500 g), resuspended in ice-cold lysis buffer (25 mM Tris-HCl pH 7.2, 150 mM NaCl, 0.2% NP-40, protease inhibitor cocktail (Sigma), 3 mM PMSF) lysed and cleared by centrifugation (20 min, 17,000 g). 10 μg of recombinant protein for GST pull-down assay, or 10 μg of the respective antibody for all other immunoprecipitations were mixed with yeast lysates, incubated with Glutathione Sepharose 4B beads or Protein A magnetic beads for at least 30 min, washed, and samples were separated on NuPage Bis-Tris gels (Life Technologies) prior to immunoblotting.

### Pho8ΔN60 assays

Pho8-activity was determined as previously described [37,38]. Briefly, 4 OD_600_ yeast cells were lysed and lysates were incubated with 50 μl 55 mM α-naphtyl phosphate disodium salt. Reactions were quenched after 20 min and fluorescence was recorded at 345/472 nm and normalized to total protein content in lysates.

### Expression and purification of proteins

Atg32, Atg9, Atg17, Atg29 and Atg31 were expressed and purified as previously described [11,32,39]. Atg11 or its N-terminal fragment Atg11^ΔC^ were coexpressed from pCoofy37-Atg11 or pCoofy37-Atg11^ΔC^ vectors with chaperones from pG-KJE8 (Takara) in *Escherichia coli* BL21 (DE3). Expression was induced with 0.3 mM Isopropyl β-D-1-thiogalactopyranoside (IPTG) for 18 h. Atg11 was purified by Ni-NTA-affinity chromatography followed by PreScission protease digestion and gel filtration (Superose6 Increase 10/300 (GE Healthcare) or Superdex200 16/60 columns). Protein aliquots were stored at −80 °C until use.

### Atg9-liposome preparation and floatation assay

Atg9^core^ was reconstituted in liposomes by rapid dilution as previously described [11] from mixtures of synthetic lipids containing 20 mol% cholesterol, 10 mol% POPE, 60 mol% POPC, and 10 mol% POPS. Liposomes were resolubilized with LDAO and Atg9^core^ (protein:lipid ratio of 1:200) was added. The mixture was 30-fold diluted and Atg9-PLs were collected by ultra-centrifugation and resuspended in interaction buffer. For cofloatation experiments, Atg9-PLs were mixed with Nycodenz and interaction partners and a step gradient of 40% Nycodenz-Atg9-PLs, 30% Nycodenz, and buffer was prepared. After ultracentrifugation, the floated fraction containing accumulated Atg9-PLs and interaction partners was analyzed by SDS-PAGE.

### Biophysical experiments

ESI-TOF mass spectrometry was performed in LC-MS mode on a BRUKER microTOF mass spectrometer and sedimentation velocity in an Optima XL-I analytical ultracentrifuge with an An-60 Ti rotor (Beckman-Coulter). Data were evaluated with SEDFIT software package [40]. Dynamic light scattering was performed as described previously [11].

### Cryo-electron microscopy

Atg9-PLs were cosonicated with Atg11 and mixed with Atg32^SE^ or buffer. Samples were added on a glow discharged Lacey carbon grid (Agar Scientific), blotted and plunge-frozen in liquid ethane using a Leica EM GP system. Samples were visualized on a Tecnai F20 electron microscope operating at a voltage of 200 kV. Images were recorded in low-dose mode on a Falcon II direct electron detector (FEI).

### Cross-linking

Atg32^SE^ was mixed with a 10x molar excess of Sulfo-LC-SDA (sulfosuccinimidyl 6-(4,4'-azipentanamido)hexanoate, Thermo Fisher), incubated for 20 min at room temperature and quenched with 100 mM final concentration of Tris pH 8.0. Residual crosslinker was removed using a Zeba spin desalting column (Thermo Fisher). Labeled Atg32^SE^ was mixed with Atg11 and exposed to 365 nm UV light for up to 15 min.

### Reconstitution on giant unilamellar vesicles

GUVs were prepared by electroformation [41] using synthetic lipid mixtures containing POPC (39.5 mol%), POPS (20 mol%), POPE (20 mol%), cholesterol (20 mol%), and Atto633-labeled POPE (0.5 mol%). Single cysteine mutants of Atg8^ΔR117^, Atg7, Atg3, Atg12–Atg5, and Atg16 were produced as described and incubated with GUV-suspensions in Lab-Tek observation chambers at a molar ratio of 6:2:2:1:1 for 1 h at 30 °C.

### Confocal microscopy

The confocal imaging was performed at the Imaging Facility of Max Planck Institute of Biochemistry, Martinsried, on a LEICA (Wetzlar, Germany) TCS SP8 AOBS confocal laser scanning microscope equipped with a LEICA HC PL APO 63x/NA1.4 oil immersion objective or on a ZEISS (Jena, Germany) LSM780 confocal laser scanning microscope equipped with a ZEISS Plan APO 63x/NA1.46 oil immersion objective. Leica LAS AF and Zeiss ZEN 2011 software packages were used, respectively. Image analysis was performed manually by using ImageJ or automated by employing a custom-made Fiji script.

## Supporting information

S1 Fig Selective and nonselective functions of Atg11 and Atg17. (A) The number of Atg11 puncta per cell was assessed in WT and *atg17Δ* cells during vegetative growth and under starvation. Atg11 was expressed using its wildtype promoter and was fused to a tandem GFP-tag. (B) Quantification of WT and *atg17Δ* cells showing puncta positive for both Atg11 and Atg8 during vegetative growth and under starvation. Atg11 was expressed using its wildtype promoter and was fused to a tandem GFP-tag. (A, B) Quantification of flattened z-stacks of >160 cells per condition was performed using a Fiji script. (C) Cytoplasmic Pho8Δ60 (cytPho8) assay of log-phase growing and 4 h starved WT, *atg5Δ* (negative control), *atg11Δ*, *atg17Δ*, and *atg11Δatg17Δ* cells as indicated. Pho8-activity was corrected for total protein amount of each sample and normalized to the signal of WT cells growing in vegetative conditions (set to 100%). (D) Mitochondrial Pho8Δ60 (mtPho8) assay of log-phase growing, and 4 h or 24 h starved WT, *atg11Δ*, *atg17Δ*, and *atg11Δatg17Δ* cells. Pho8-activity was corrected with total protein amount of each sample and normalized to the signal of WT cells growing in vegetative conditions (set to 100%). (C, D) Data are presented as mean values ± SD of *n* = 3 independent experiments. Related to Fig 1.
(TIF)

S2 Fig Characterization of recombinant Atg11 in vitro and degradation of Atg11 in vivo. (A) UV and Total Ion Current (TIC) chromatogram of ESI-TOF mass spectrometric analysis of recombinantly expressed and purified Atg11, containing a C-terminal His_6_-tag and N-terminal residual glycine-proline from PreScission protease digestion. (B) Elution profile of purified Atg11-His_6_ from SEC on a Superose6 Increase 10/300 column. (C) Sedimentation velocity analysis of purified Atg11-His_6_ by analytical ultracentrifugation. Data were analyzed using SEDFIT and the sedimentation coefficient (S) distribution is shown. (D) SDS-PAGE gel with input (100% of protein used for cofloatation) and floated fractions from cofloatation assay of Atg9-PLs with Atg11, Atg17, and Atg17^TC^ (trimeric complex of Atg17, Atg31 and Atg29). SUVs lacking Atg9^core^ served as control for unspecific membrane binding. (E) SDS-PAGE gel of pull-down assay using 10 μg recombinant GST-tagged Atg31 (31) and Atg29^WT^ (29) or Atg29^SD^ (29^SD^, coexpressed and purified as subcomplex) with recombinant Atg11. GST served as negative control. (F) Western blots of lysates from wildtype cells that expressed myc-Atg17 (genomically tagged) using anti-myc antibodies. Pgk1 served as loading control. The chart shows quantification of blots from three independent experiments, normalized to the amount of Pgk1, relative to the amount in nonstarved cells, which was set to 1. Data are presented as mean values ± SD. (G) Analysis of Atg11 protein levels in nonstarved and starved cells in which the vacuolar hydrolase pep4 was deleted. Atg11-HA protein levels in lysates were detected by immunoblotting using anti-HA antibodies. The corresponding charts show quantification of band intensities from whole cell extracts. (H) Ubiquitination of Atg11 was identified by IP from lysates of myc-Atg11 expressing cells using protein A magnetic beads charged with anti-Ub antibodies. Atg11 was detected using anti-myc western blotting of lysates and fractions from immunoprecipitations of starved and nonstarved cells. Lysates of nontagged *atg11* served as control. The chart shows quantification of band intensities from immunoprecipitated fractions as shown in the corresponding blot (Ub-IP). Data are presented as mean values ± SD of *n* = 3 independent experiments. *P* values were calculated using a two-tailed Student *t* test **P < 0.01). (I) Analysis of Atg11 protein levels in nonstarved and starved cells treated with the proteasomal inhibitor MG132 (DMSO as control) Atg11-HA protein levels in lysates were detected by immunoblotting using anti-HA antibodies. The corresponding charts show quantification of band intensities from whole cell extracts. Data are presented as mean values ± SD of *n* = 4 (G) and *n* = 3 (I) independent experiments. Related to Fig 3.
(TIF)

S3 Fig The Atg17-like domain of Atg11 is required for dimerization. (A) Scheme to visualize the domain architecture and domain borders of Atg11 and its fragments Atg11^Δ^ and Atg11^Δdim^. Predicted coiled-coil regions are indicated in grey. (B) Sedimentation velocity analysis of Atg11^ΔC^ and Atg11 at high (grey lines) and low (black lines) concentrations by analytical ultracentrifugation. The sedimentation coefficient (S) distributions that best fit the data as analyzed by SEDFIT are shown. Atg11^ΔC^ was used at 6.47 mg/ml (high conc.) and 0.19 mg/ml (low conc.), Atg11 at 1.63 mg/ml (high conc.) and 0.15 mg/ml (low conc.). (C) IP of GFP-tagged Atg11 or its fragments Atg11^ΔC^ and Atg11^Δdim^ and co-IP of HA-tagged Atg9 from lysates of log-phase growing and 2 h starved cells. Lysate of nontagged *atg11* cells served as control. Atg11-variants and Atg9 were detected by α-GFP and α-HA immunoblots, respectively. (D) Mitochondrial Pho8ΔN60 (mtPho8) assay of log-phase growing and starved cells expressing Atg11^WT^, Atg11^ΔC^, or Atg11^Δdim^. Pho8 activity was corrected for total protein amount of each sample and normalized to the signal of log-phase WT cells (= 1). Data are presented as mean values ± SD of *n* = 3 independent experiments. (E) Cytoplasmic Pho8ΔN60 (cytPho8) assay of log-phase growing and starved wildtype and *atg17Δ* cells that expressed Atg11 or its fragments as indicated. The Pho8 activity was corrected with total protein amount of each sample and normalized to the signal of nonstarved WT cells that expressed Atg11 (set to 100%). Data are presented as mean values ± SD of *n* = 3 independent experiments. *P* values were calculated using a two-tailed Student’s t-test (*P < 0.05, **P < 0.01, ***P < 0.001). Related to Fig 4.
(TIF)

S4 Fig Atg11 physically interacts with Atg8 and Atg32. (A) PacificBlue-labeled Atg32-variants Atg32^SE^ (phosphomimetic Atg32) and Atg32^WG^ (disrupted Atg8-interacting motif) were tested for their interaction with Atg8^Atto488^, enzymatically coupled to GUVs. (B) Recombinant Atg11^Atto565^ was added to Atto633-labeled GUVs in the presence or absence of Atg8^Atto488^, which has been enzymatically conjugated to GUV-membranes. (C) Atto488-labeled Atg32^SE,WG^ was added to Atto633-labeled GUVs decorated with Atg8^PacificBlue^ and Atg11^Atto565^. Membranes covered with Atg8 but devoid of Atg11 served as internal control. Atg32^SE,WG^ is not recruited to membranes in the absence of Atg11 (arrowhead). (A–C) Colors of Atg8 and Atg32 were assigned as used in other figures, not according to the dye they were labeled with. Scale bars 10 μm. Related to Fig 5.
(TIF)

S5 Fig Original blots and SDS gels. Source data for blots and gels. Original gels and blots with cropped areas shown in Figures of the manuscript and Supplemental Information as indicated. The cropped areas are indicated by frames. Related to Figs 2, 3 and 4, S2 and S3 Figs.
(TIF)

S1 Table Yeast strains used in this study. (XLSX)

S2 Table Plasmids used in this study. (XLSX)

S3 Table Yeast strains and plasmids used in figures. (XLSX)

S1 Text Supporting methods and references. (DOCX)

S1 Data Source data for quantification presented as charts with measured values and calculated means, standard deviations, as well as statistical significance analysis. (XLSX)

## Abbreviations

AIM: Atg8-interacting motif
Atg1^PC^: pentameric Atg1 kinase complex
Atg9-PL: Atg9-proteoliposome
DLS: dynamic light scattering
EM: electron microscopy
GFP: green fluorescent protein
GST: glutathione-S-transferase
GUV: giant unilamellar vesicle
HA: Hemagglutinin
IP: immunoprecipitation
PAS: phagophore assembly site
R_H_: hydrodynamic radius
SUV: small unilamellar vesicle
Ub: ubiquitin
