# Supplementary figures and images for: Atg11 tethers Atg9 vesicles to initiate selective autophagy
Source: PLoS Biol. 2019 Jul 29;17(7):e3000377. doi: 10.1371/journal.pbio.3000377 (PMC6687201; doi:10.1371/journal.pbio.3000377)

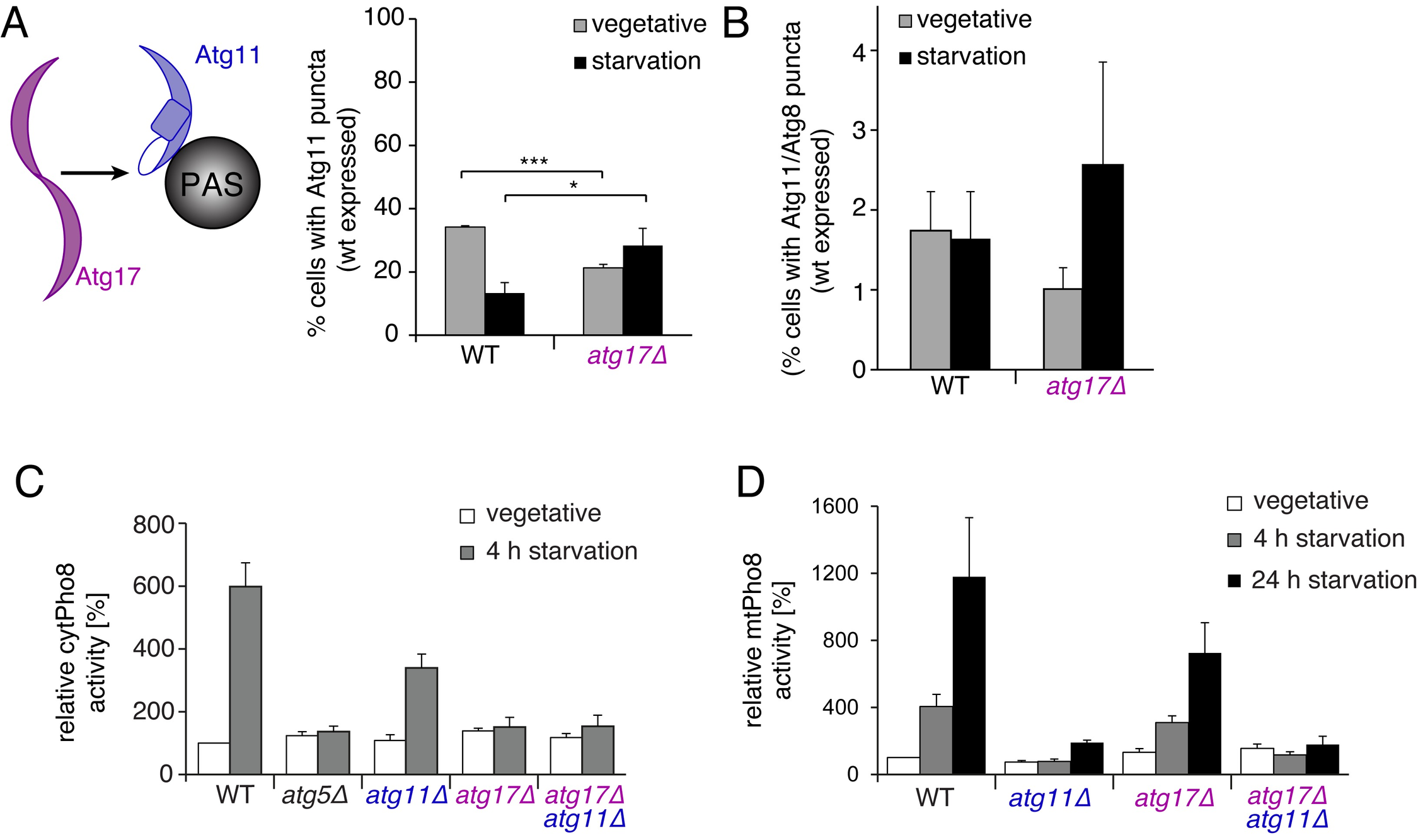

Supplement: S1 Fig — (A) The number of Atg11 puncta per cell was assessed in WT and atg17Δ cells during vegetative growth and under starvation. Atg11 was expressed using its wildtype promoter and was fused to a tandem GFP-tag. (B) Quantification of WT and atg17Δ cells showing puncta positive for both Atg11 and Atg8 during vegetative growth and under starvation. Atg11 was expressed using its wildtype promoter and was fused to a tandem GFP-tag. (A, B) Quantification of flattened z-stacks of >160 cells per condition was performed using a Fiji script. (C) Cytoplasmic Pho8Δ60 (cytPho8) assay of log-phase growing and 4 h starved WT, atg5Δ (negative control), atg11Δ, atg17Δ, and atg11Δatg17Δ cells as indicated. Pho8-activity was corrected for total protein amount of each sample and normalized to the signal of WT cells growing in vegetative conditions (set to 100%). (D) Mitochondrial Pho8Δ60 (mtPho8) assay of log-phase growing, and 4 h or 24 h starved WT, atg11Δ, atg17Δ, and atg11Δatg17Δ cells. Pho8-activity was corrected with total protein amount of each sample and normalized to the signal of WT cells growing in vegetative conditions (set to 100%). (C, D) Data are presented as mean values ± SD of n = 3 independent experiments. Related to Fig 1. (TIF) [file pbio.3000377.s001.tif]

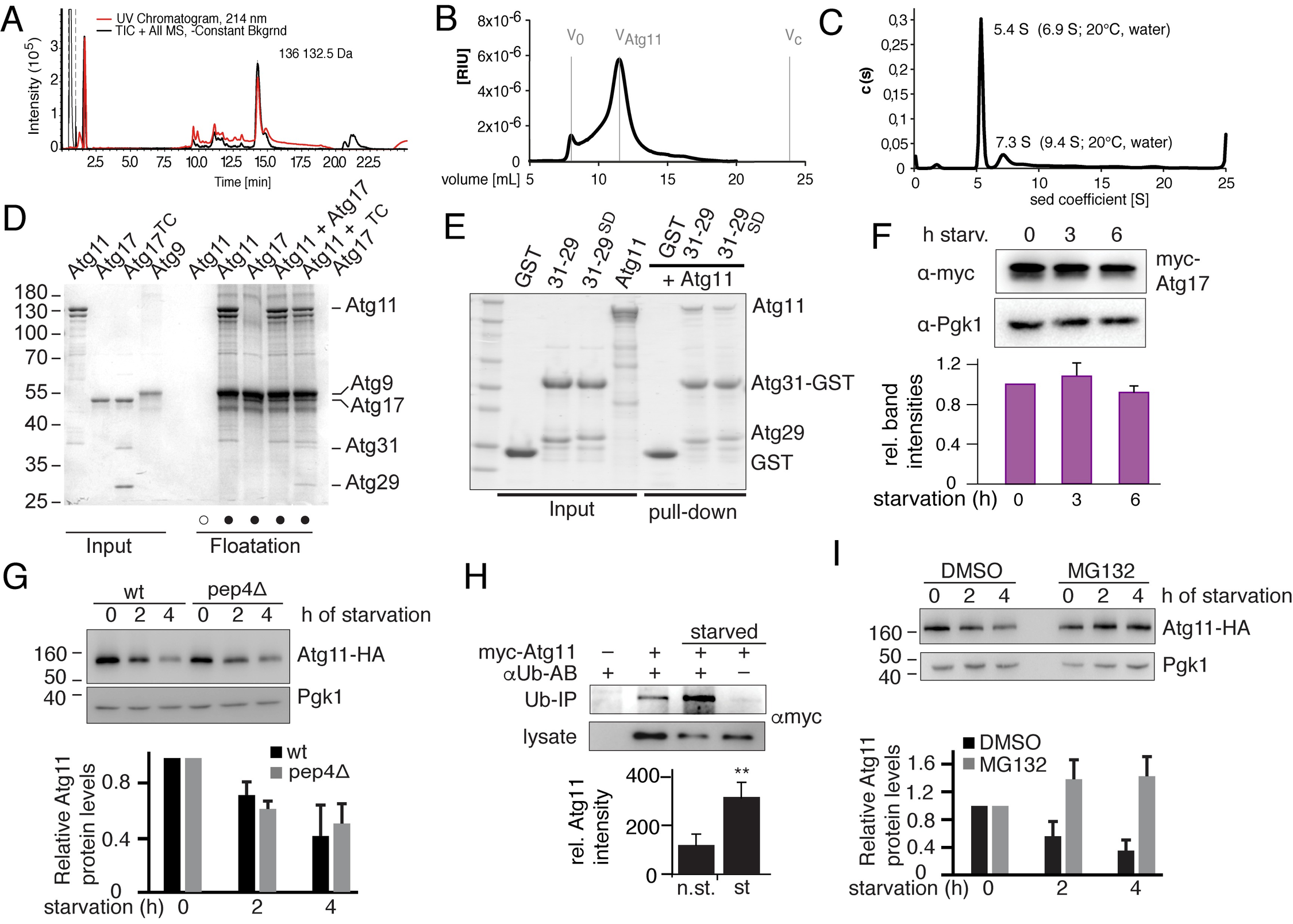

Supplement: S2 Fig — (A) UV and Total Ion Current (TIC) chromatogram of ESI-TOF mass spectrometric analysis of recombinantly expressed and purified Atg11, containing a C-terminal His6-tag and N-terminal residual glycine-proline from PreScission protease digestion. (B) Elution profile of purified Atg11-His6 from SEC on a Superose6 Increase 10/300 column. (C) Sedimentation velocity analysis of purified Atg11-His6 by analytical ultracentrifugation. Data were analyzed using SEDFIT and the sedimentation coefficient (S) distribution is shown. (D) SDS-PAGE gel with input (100% of protein used for cofloatation) and floated fractions from cofloatation assay of Atg9-PLs with Atg11, Atg17, and Atg17TC (trimeric complex of Atg17, Atg31 and Atg29). SUVs lacking Atg9core served as control for unspecific membrane binding. (E) SDS-PAGE gel of pull-down assay using 10 μg recombinant GST-tagged Atg31 (31) and Atg29WT (29) or Atg29SD (29SD, coexpressed and purified as subcomplex) with recombinant Atg11. GST served as negative control. (F) Western blots of lysates from wildtype cells that expressed myc-Atg17 (genomically tagged) using anti-myc antibodies. Pgk1 served as loading control. The chart shows quantification of blots from three independent experiments, normalized to the amount of Pgk1, relative to the amount in nonstarved cells, which was set to 1. Data are presented as mean values ± SD. (G) Analysis of Atg11 protein levels in nonstarved and starved cells in which the vacuolar hydrolase pep4 was deleted. Atg11-HA protein levels in lysates were detected by immunoblotting using anti-HA antibodies. The corresponding charts show quantification of band intensities from whole cell extracts. (H) Ubiquitination of Atg11 was identified by IP from lysates of myc-Atg11 expressing cells using protein A magnetic beads charged with anti-Ub antibodies. Atg11 was detected using anti-myc western blotting of lysates and fractions from immunoprecipitations of starved and nonstarved cells. Lysates of nontagged atg11 [file pbio.3000377.s002.tif]

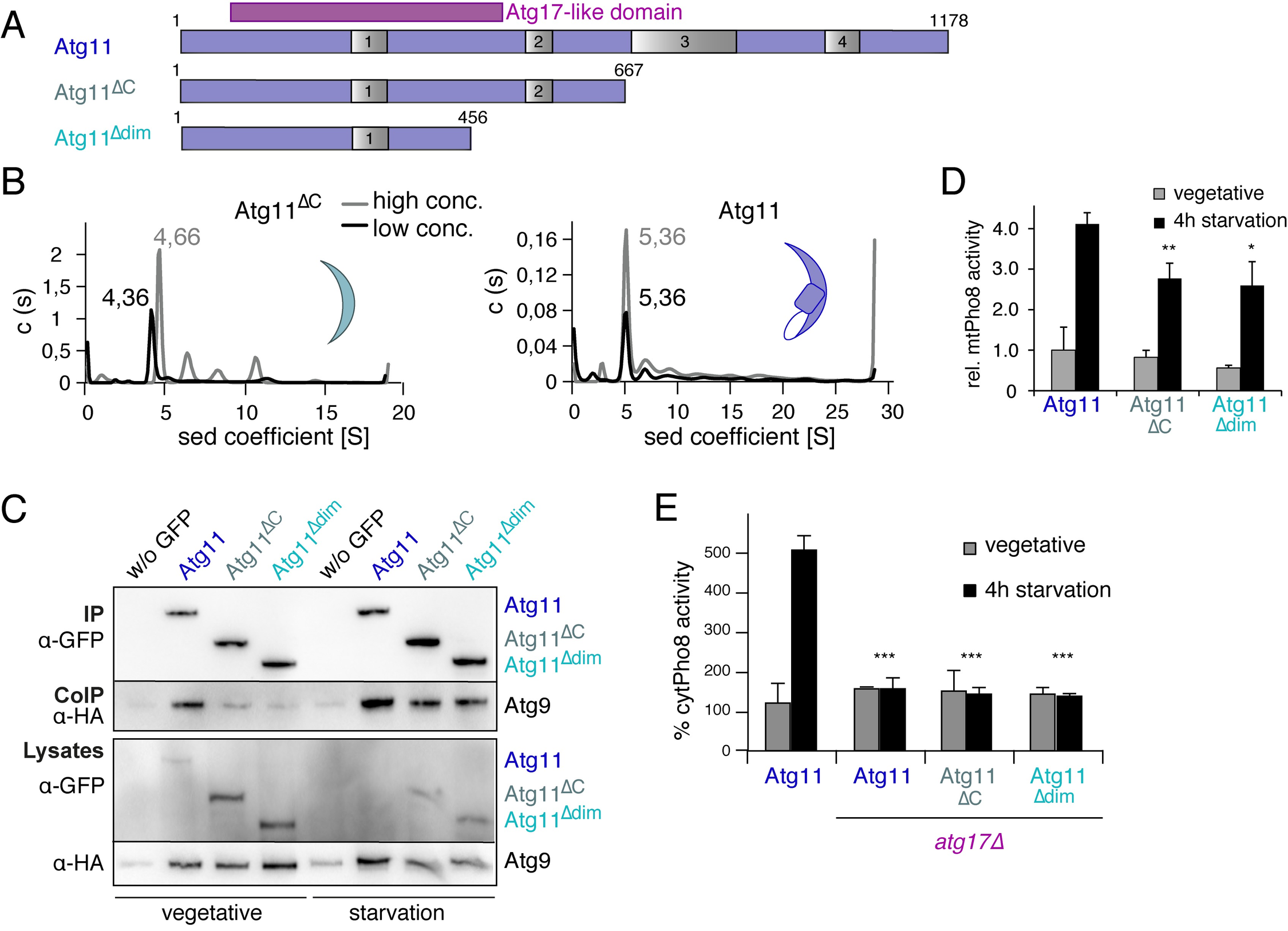

Supplement: S3 Fig — (A) Scheme to visualize the domain architecture and domain borders of Atg11 and its fragments Atg11Δ and Atg11Δdim. Predicted coiled-coil regions are indicated in grey. (B) Sedimentation velocity analysis of Atg11ΔC and Atg11 at high (grey lines) and low (black lines) concentrations by analytical ultracentrifugation. The sedimentation coefficient (S) distributions that best fit the data as analyzed by SEDFIT are shown. Atg11ΔC was used at 6.47 mg/ml (high conc.) and 0.19 mg/ml (low conc.), Atg11 at 1.63 mg/ml (high conc.) and 0.15 mg/ml (low conc.). (C) IP of GFP-tagged Atg11 or its fragments Atg11ΔC and Atg11Δdim and co-IP of HA-tagged Atg9 from lysates of log-phase growing and 2 h starved cells. Lysate of nontagged atg11 cells served as control. Atg11-variants and Atg9 were detected by α-GFP and α-HA immunoblots, respectively. (D) Mitochondrial Pho8ΔN60 (mtPho8) assay of log-phase growing and starved cells expressing Atg11WT, Atg11ΔC, or Atg11Δdim. Pho8 activity was corrected for total protein amount of each sample and normalized to the signal of log-phase WT cells (= 1). Data are presented as mean values ± SD of n = 3 independent experiments. (E) Cytoplasmic Pho8ΔN60 (cytPho8) assay of log-phase growing and starved wildtype and atg17Δ cells that expressed Atg11 or its fragments as indicated. The Pho8 activity was corrected with total protein amount of each sample and normalized to the signal of nonstarved WT cells that expressed Atg11 (set to 100%). Data are presented as mean values ± SD of n = 3 independent experiments. P values were calculated using a two-tailed Student’s t-test (*P < 0.05, **P < 0.01, ***P < 0.001). Related to Fig 4. (TIF) [file pbio.3000377.s003.tif]

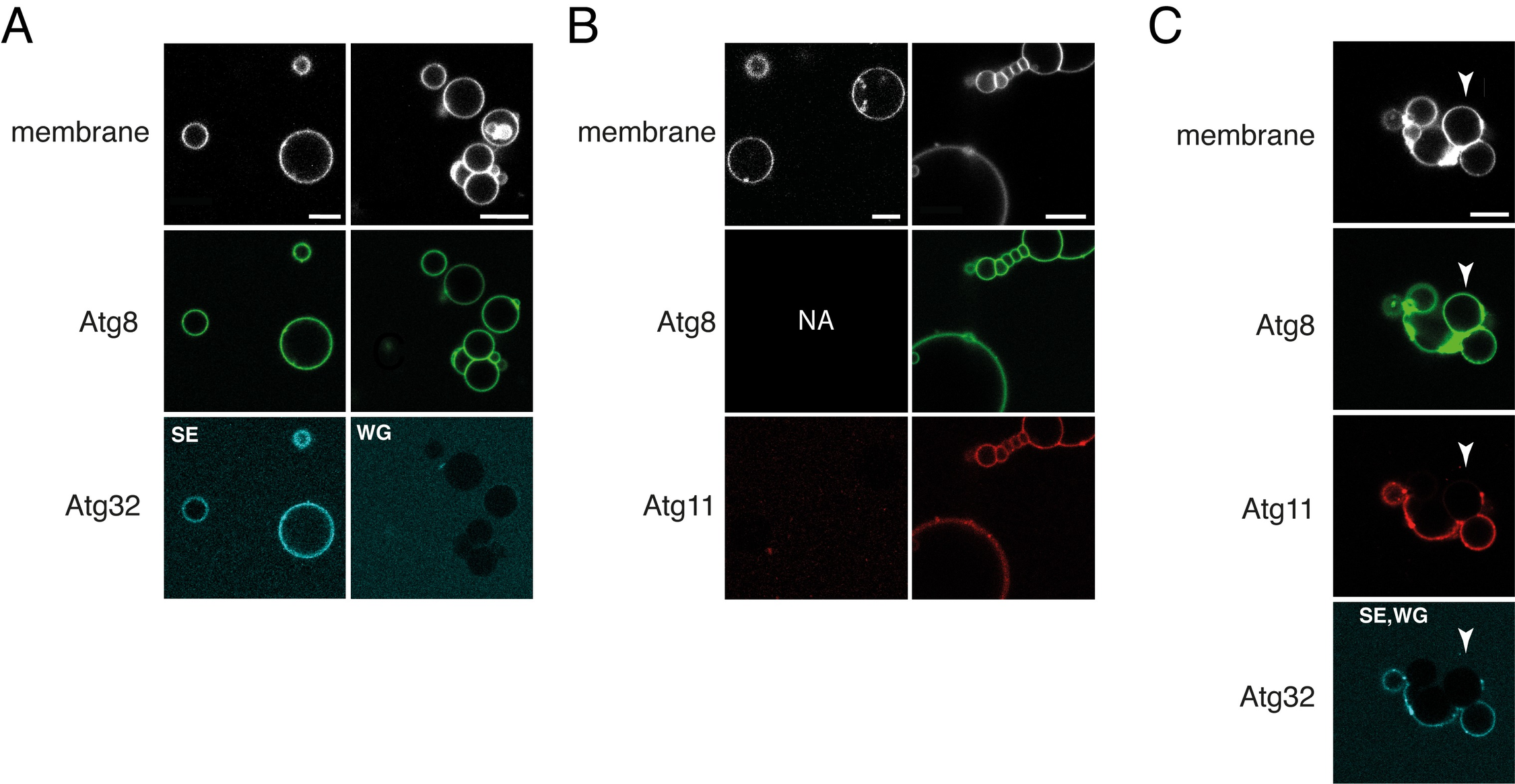

Supplement: S4 Fig — (A) PacificBlue-labeled Atg32-variants Atg32SE (phosphomimetic Atg32) and Atg32WG (disrupted Atg8-interacting motif) were tested for their interaction with Atg8Atto488, enzymatically coupled to GUVs. (B) Recombinant Atg11Atto565 was added to Atto633-labeled GUVs in the presence or absence of Atg8Atto488, which has been enzymatically conjugated to GUV-membranes. (C) Atto488-labeled Atg32SE,WG was added to Atto633-labeled GUVs decorated with Atg8PacificBlue and Atg11Atto565. Membranes covered with Atg8 but devoid of Atg11 served as internal control. Atg32SE,WG is not recruited to membranes in the absence of Atg11 (arrowhead). (A–C) Colors of Atg8 and Atg32 were assigned as used in other figures, not according to the dye they were labeled with. Scale bars 10 μm. Related to Fig 5. (TIF) [file pbio.3000377.s004.tif]

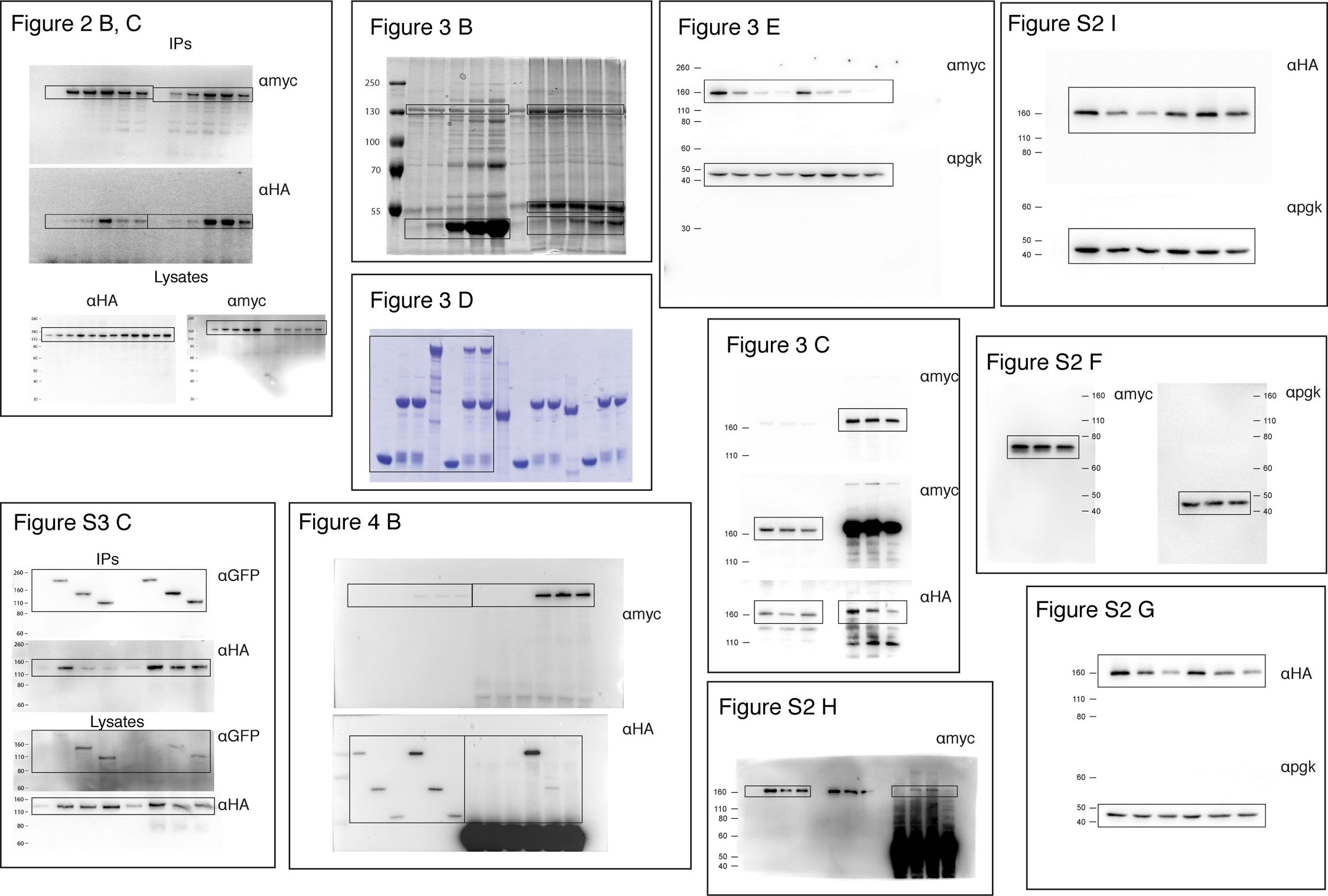

Supplement: S5 Fig — Source data for blots and gels. Original gels and blots with cropped areas shown in Figures of the manuscript and Supplemental Information as indicated. The cropped areas are indicated by frames. Related to Figs 2, 3 and 4, S2 and S3 Figs. (TIF) [file pbio.3000377.s005.tif]
