# Supplementary material for: Atg11 tethers Atg9 vesicles to initiate selective autophagy
Source: PLoS Biol. 2019 Jul 29;17(7):e3000377. doi: 10.1371/journal.pbio.3000377 (PMC6687201; doi:10.1371/journal.pbio.3000377)
Supplement: S1 Text — (DOCX) [file pbio.3000377.s009.docx]

# Supporting Methods

## Yeast strains and growth conditions

Derivatives of *S. cerevisiae* BY4741 and BY4742 (Euroscarf) used for each experiment of this study are listed in Supplemental Table 3 with genotypes specified in Supplemental Table 1. Genomic gene deletion and tagging was performed as described previously [1]. Yeast cells were grown at 30°C in YPD media (1% yeast extract, 2% peptone, 2% glucose) or in synthetic dropout media (0.67% yeast nitrogen base, 2% glucose) supplemented with dropout amino acid mix. For growth on plates, 2% agar was added to the respective media. Autophagy was induced by harvesting cultures grown to early log phase, washing and resuspending them in SD-N media (0.17% yeast nitrogen base, 2% glucose) for at least 2 h.

## Cloning of plasmids

Plasmids used in this study are listed in Supplementary Table 2. Live-cell imaging of Atg11, Atg17, and Atg9 in yeast, co-IPs and Pho8-assays were performed with proteins expressed from centromere-based vectors pTL58 (LEU2 selection if not noted otherwise) or pRS316 (URA3 selection) with native promoters for endogenous levels or pPma1 promoters for overexpression; mCherry-tagged Atg8 was expressed from pMet25-inducible pUG36. Point mutations in *atg29* and atg*32* were introduced by employing the QuikChange Lightning Site-Directed Mutagenesis Kit (Agilent). Cloning of plasmids for in vivo assays or protein expression was performed with coding DNA amplified from BY4741 or previously used plasmids according to the published method based on Seamless Ligation Independent Cloning (SLIC) [2] if not noted otherwise.

## Preparation of yeast cell extracts and immunoprecipitation

Lysates for immunoprecipitation of myc-tagged proteins were prepared from 50 OD_600_ units for each sample of log phase growing or 2 h starved cells. Cells were washed in lysis buffer (25 mM Tris pH 7.2, 150 mM NaCl, 200 mM sorbitol, 1 mM MgCl_2_, 0.1% Tween-20), resuspended in 500 µl lysis buffer supplemented with 20 µl protease inhibitor cocktail (Sigma-Aldrich) and 3 mM PMSF and disrupted by shearing with glass beads. Cellular debris was removed by centrifugation for 2 min at 6000 rpm, pre-cleared lysates were supplemented with Tween-20 (0.9% final concentration) for 10 min at 4°C and centrifuged at 14000 rpm for 10 min at 4°C. Lysates were diluted with 500 µl lysis buffer and 10 µg mouse α-myc antibody (in-house) were added and incubated for 2 h at 4°C. For each sample, 40 µl of Protein A magnetic beads (New England Biolabs) were washed in TBS-T (25 mM Tris pH 7.5, 150 mM NaCl, 0.05% Tween-20) and incubated with lysate-antibody for 30 min. Beads were washed twice with lysis buffer, directly resuspended in 2x SDS sample buffer and boiled for 10 minutes. For immunoprecipitation of ubiquitinylated proteins 500 OD_600_ units were resuspended in 3 ml of lysis buffer supplemented with 150 µl protease inhibitor cocktail and 3 mM PMSF. The sample was processed as described before, except that it was incubated with 20 µg α-ub antibody (mono- and polyubiquitinylated conjugates; Enzo Life Sciences) followed by incubation with 50 µl of washed Protein A magnetic beads (Thermo Fisher).

**Competition of purified Atg17 and endogenous Atg11 for Atg9-binding in vivo**

Lysates from 120 OD of cells expressing HA-tagged Atg9 and myc-tagged Atg11 were each incubated with 50 or 200 µg recombinant Atg17 or buffer for 30 min at 4°C. Subsequent immunoprecipitation using α-myc antibody was performed as described above.

**Proteasome inhibition and whole cell extracts**

For proteasome inhibition pdr5Δ knockout strains were grown to log phase and switched to SD-N starvation medium supplemented with 75 µM (S)-MG132 (Cayman Chemical) or an equal volume of DMSO. For quantifying relative protein levels by Western blotting, whole cell extracts were prepared. 1 OD of cells was incubated with 2.0 M lithium acetate followed by an incubation with 0.4 M NaOH for 5 min on ice each. The cells were boiled for 5 min in 1x SDS sample buffer and debris was removed by centrifugation prior to gel electrophoresis.

## Alkaline phosphatase activity measurements

Measurements of cytoplasmic and mitochondrial targeted alkaline phosphatase (Pho8) activity were used to quantify autophagy and mitophagy, respectively, and performed as described previously [2,3]. Briefly, lysates of 4 OD_600_ units of cells growing in early log phase or starved for the indicated time were prepared by shearing with glass beads in 400 µl assay buffer (250 mM Tris pH 9.0, 10 mM MgSO_4_, 10 µM ZnSO_4_). 50 µl of each lysate were diluted in 450 µl assay buffer and 50 µl 55 mM α-naphtyl phosphate disodium salt was added. Enzymatic reaction was stopped after 20 min at 30°C with 500 µl of 2 M glycine pH 11.0 and fluorescence was recorded at 345/472 nm in a 96-well plate (plate reader BioTek Synergy Neo). Signal was normalized to protein content of lysates, determined by a BCA assay (Pierce BCA Protein Kit, Thermo).

**Quantification of band intensities of western blots and SDS-gels and statistical analysis.**

For quantitative analysis of protein levels in western blots or SDS-gels, yeast cells were freshly transformed. For experiments related to Figure 2, cultures of the corresponding independent experiments were grown and processed independently but parallel to minimize the impact of variation of experimental or environmental conditions. Western blots were developed using HRP-conjugated secondary antibodies followed by incubation with ECL substrate (Supersignal West Pico Plus; Thermo Fisher) and imaging in a gel documentation system. All band intensities were quantified using ImageJ by integrating the grey values of the bands and subtracting the corresponding background grey levels. All samples were normalized using indicated protein standards (Pgk1 in cell lysates and input levels in co-IP experiments) and expressed as relative values by setting the corresponding reference values to 1.0 (during starvation: non starved condition, in competition experiments with purified Atg17: no Atg17 addition, for Atg11-self interaction: Atg11 full-length).

Statistical significance was tested using two-tailed Student’s t-test with the indicated confidence levels.

## Recombinant expression and purification of proteins

The open reading frames (ORFs) encoding Atg9, Atg17, Atg29 and Atg31 were amplified from yeast S. cerevisiae BY4741 (Euroscarf) genomic DNA. Atg17, Atg29-Atg31, Atg29^3SD(3SA)^-Atg31 and Atg17-Atg31-Atg29 complex were (co)expressed from either the polycistronic vector pST39 with a His_6_-tag fused to the N-terminus of Atg17, or from pGEX6P-1 with a GST-tag fused to the C-terminus of Atg31 in E. coli Rosetta cells. Atg29^3SD^ was expressed as GST-fusion protein from the pGEX6P-1 vector. The coding sequence of Atg9^core^ (Atg9^281-779^) was sub-cloned into a modified pET28a(+)vector (Novagen). All Atg-proteins were expressed and purified as described [4]. Recombinant protein expression of Atg11 or its N-terminal fragment Atg11^ΔC^ was performed from pCoofy37-Atg11 and pCoofy37-Atg11^ΔC^ vectors and co-expressed with chaperones from pG-KJE8 (Takara) in *E. coli* BL21(DE3). Cultures were grown to an OD_600_ of one at 37°C, cooled down to 18°C for 2 h and induced with 0.3 mM Isopropyl β-D-1-thiogalactopyranoside (IPTG) for 18 h. Cells were lysed using a Microfluidizer and cellular debris was removed by centrifugation for 1 h at 50,000 g. Atg11 was purified by Ni-NTA-affinity chromatography using HisTrap FF Crude (GE Healthcare) column. MBP-Atg11^ΔC^ was purified using 1 ml Ni-NTA agarose (QIAGEN). MBP-fusion proteins were digested by PreScission protease in the presence of 1 mM DTT and 1 mM EDTA for 15 min at room temperature. The protein was further purified by gelfiltration using Superose6 Increase 10/300 (GE Healthcare) (Atg11) or a Superdex200 16/60 (Atg11^ΔC^) column equilibrated with Tris buffer pH 7.2, 275 mM KCl, 5% glycerol. Recombinant Atg32^1-376^ and respective mutants were expressed in *E.coli* Rosetta (DE3) grown to OD_600_ = 0.5 and expression overnight was induced by adding 0.3 mM IPTG. Cultures were harvested, resuspended in lysis buffer (100 mM Tris pH 8.0, 300 mM NaCl, 5 mM imidazole, 10% glycerol, 5 mM β-mercaptoethanol) and lysed by sonication. Cell lysate was cleared by centrifugation at 50,000 g and the supernatant was incubated with Ni-NTA agarose resin for 1 h at 4°C. Beads were washed with 500 ml wash buffer (100 mM Tris pH 7.2, 300 mM NaCl, 5 mM imidazole, 10% glycerol, 5 mM β-mercaptoethanol) and protein was eluted with 5 ml elution buffer (50 mM Tris pH 7.2, 300 mM NaCl, 250 mM imidazole, 10% glycerol). The affinity-tag was cleaved from the protein by PreScission protease digestion. Atg32 was further purified by gelfiltration using a Superdex200 16/600 (GE Healthcare) column with 25 mM Tris pH 7.2, 275 mM NaCl as running buffer. Protein aliquots were used freshly or frozen in liquid N_2_ and stored at -80°C until use.

## GST pull-downs

For pull-downs of GST-tagged proteins, 10 μg of GST, GST-Atg29^SD^, or Atg29^SD^-Atg31-GST were mixed with 20 μg recombinant Atg11, incubated with Glutathione Sepharose 4B beads for 2 hours, extensively washed with lysis buffer containing an additional 100 mM NaCl and resuspended in SDS sample buffer. Samples were separated on precast NuPage 4-12% Bis-Tris gradient gels (Novex, Thermo).

## Reconstitution of Atg9 in liposomes and floatation assay

Atg9^core^ was reconstituted in liposomes by rapid dilution as previously described [4]. Briefly, mixtures of synthetic lipids containing 20 mol% cholesterol, 10 mol% POPE, 60 mol% POPC, and 10 mol% POPS were used to prepare liposomes, which were re-solubilized with LDAO and Atg9^core^ was added using a protein:lipid ratio of 1:200. The mixture was 30-fold diluted and Atg9^core^ containing proteoliposomes (Atg9-PLs) were harvested by ultra-centrifugation, resuspended in 25 mM HEPES pH 7.0, 100mM NaCl (interaction buffer) and extruded using a filter pore size of 200 nm. The interaction of Atg11, Atg11^ΔC^, Atg17^TC^, or Atg17^TC^ subunits with Atg9-PLs was assessed by floatation experiments. Therefore, Atg9-PLs were mixed with an equivalent volume of 80% Nycodenz containing the respective interaction partners in a stoichiometric ratio of 1:1. A step gradient using 30% Nycodenz and buffer was prepared by overlaying 250 µl of each of the Nycodenz solutions with 100 µl interaction buffer. After centrifugation at 165,000 g for 2 h (at 4°C), the floated fraction containing accumulated Atg9-PLs and interaction partners was analyzed by SDS-PAGE and immunoblotting.

## Mass spectrometry and Analytical ultracentrifugation

Biophysical characterization of recombinant Atg11 was performed by the Biochemistry Core Facility of the MPI-B. ESI-TOF mass spectrometry was performed in LC-MS mode on a BRUKER microTOF mass spectrometer. Sedimentation velocity of Atg11 and Atg11^ΔC^ was determined by analytical ultracentrifugation (AUC) in an Optima XL-I analytical ultracentrifuge with an An-60 Ti rotor (Beckman-Coulter). Centrifugation was performed at 50,000 rpm, 20°C and sedimentation was scanned by absorbance at 280 nm. Density and viscosity of the protein buffers used were determined with a DMA 5000 density meter and an AMVn viscometer, respectively (Anton Paar GmbH). Fitting of sedimentation data was performed with SEDFIT software package to provide a c(s) distribution of molecules with distinct sedimentation coefficients (S), R_H_ and friction factors [5].

## Dynamic light scattering

The hydrodynamic radius of samples was determined by dynamic light scattering using a DynaPro NanoStar Instrument (Wyatt) with 50-μl cuvettes and a total sample volume of 20 μl. Proteins and Atg9–PLs were mixed using a stoichiometric ratio of 1:1 and taking Atg9-orientation in liposomes (50% of total Atg9 accessible) into account. Mixtures were prepared in buffer containing 25 mM HEPES pH 7.0 and 100 mM NaCl, incubated at room temperature for 15 min, and sonicated at 4 °C until the solution appeared clear (15 min). Measurements were carried out at 4 °C. Raw data were analyzed by the DYNAMICS software package. Sample-dispersity and the average hydrodynamic radius (R_H_) were assessed.

## Fluorescent labeling of proteins and lipid

Fluorescent dyes were purchased from Molecular Probes and dissolved in DMSO. Atg11 was labeled with amine-reactive dyes. The protein was gelfiltrated over two consecutive HiTrap desalting columns (GE Healthcare) that have been equilibrated with PBS prior labeling. Purified Atg32 and Atg8 were labeled by maleimide-coupling to native or engineered cysteine residues without desalting. The fluorescent dyes were added to the proteins yielding a molar ratio of 1:1. The mixture was incubated for 30 min at room temperature and excess of dye was removed by desalting using HiTrap desalting columns. The degree of labeling was determined spectroscopically according to the manufacturer’s protocol.

POPE was labeled at its amino-headgroup with Atto633 NHS-ester. The dye was dissolved in DMSO and deprotonated with 125 mM NaOH. POPE (1.25 mg/ml in chloroform) and dye were mixed at a 1:1 molar ratio. Labeling was allowed to proceed at room temperature for 1 h. Aliquots of labeled lipid were stored at -80°C in nitrogen atmosphere until use.

**Preparation of Giant Unilamellar Vesicles**

GUVs were produced by electroformation [6] from a lipid-mix consisting of POPC (39.5 mol%), POPS (20 mol%), POPE (20 mol%), cholesterol (20 mol%), and Atto633-labeled POPE (0.5 mol%) was prepared. A thin lipid film was spread on indium-tin oxide-covered glass slides (40 µg total lipid per slide) and dried overnight under vacuum. Two plates were placed into 5 ml 600 mM sucrose in a self-made Teflon chamber and electroformation was carried out by applying an electric AC field (1.2 V, 10 Hz) for 4 hours at 30°C. The GUVs were used within hours after harvest.

**Enzymatic coupling of Atg8 to membrane**

Expression and purification of Atg8, Atg3, Atg7, Atg12-Atg5 and Atg16 from plasmids listed in Supplementary Table 2 and enzymatic conjugation of Atg8 to GUVs was performed as described previously [7]. Briefly, all proteins were expressed in Rosetta(DE3)pLysS, except Atg7, which was expressed in BL21(DE3). Atg7 was co-expressed with chaperones from the plasmid pG-KJE8. Atg8 was constructed with an artificial N-terminal cysteine for labeling and a deleted C-terminal arginine for direct coupling to PE. Cells were lysed by sonication and proteins were purified using Ni^2+^-NTA affinity chromatography followed by SEC using running buffer (25 mM Tris pH 7.2, 275 mM NaCl). The affinity-tags were cleaved off by PreScission protease digest. Atg8^ΔR117^ was pre-incubated with Atg7 and Atg3 at a molar ratio of 3:1:1 in the presence of 0.5 mM ATP and Mg^2+^ for 30 min at 30°C. After addition of Atg12–Atg5-Atg16 (molar ratio 1/6 of Atg8), 100 µl protein solution was incubated with 100 µl GUV suspension in an observation chamber (Lab-Tek #1.0 Borosilicate) for 30 min at room temperature.

**Confocal microscopy**

Yeast live-cell imaging was performed with cells grown to mid-log phase or starved for at least 2 hours. Cells were diluted to reach an OD_600_ = 0.25 with drop-out media or SD-N in an observation chamber, which had been pre-coated with 1 mg/ml Concanavalin A (Sigma). GFP and mCherry were excited with 488-nm and 561-nm laser lines, respectively. Imaging of GUVs was performed in observation chambers pre-coated with BSA. Multi-tracking mode was used for image acquisition on the Zeiss LSM780 microscope, track 1 for excitation of PacificBlue with 405 nm and of Atto565 with 561 nm laser lines, track 2 for excitation of Atto488 with 488 nm and of Atto633 with 633 nm laser lines. All images were analyzed using ImageJ [8] with the Bio-Formats package plugin. Quantification of fluorescent puncta (except Atg9) was performed automated with a script developed by the Imaging Facility of the MPIB.

## Supporting References

1. Janke C, Magiera MM, Rathfelder N, Taxis C, Reber S, Maekawa H, et al. A versatile toolbox for PCR-based tagging of yeast genes: new fluorescent proteins, more markers and promoter substitution cassettes. Yeast. 2004;21: 947–962. doi:10.1002/yea.1142

2. Noda T, Matsuura A, Wada Y, Ohsumi Y. Novel system for monitoring autophagy in the yeast Saccharomyces cerevisiae. Biochem Biophys Res Commun. 1995;210: 126–132. doi:10.1006/bbrc.1995.1636

3. Mendl N, Occhipinti A, Müller M, Wild P, Dikic I, Reichert AS. Mitophagy in yeast is independent of mitochondrial fission and requires the stress response gene WHI2. J Cell Sci. The Company of Biologists Ltd; 2011;124: 1339–1350. doi:10.1242/jcs.076406

4. Rao Y, Perna MG, Hofmann B, Beier V, Wollert T. The Atg1-kinase complex tethers Atg9-vesicles to initiate autophagy. 2016;7: 10338. doi:10.1038/ncomms10338

5. Schuck P. Size-distribution analysis of macromolecules by sedimentation velocity ultracentrifugation and lamm equation modeling. Biophys J. 2000;78: 1606–1619. doi:10.1016/S0006-3495(00)76713-0

6. Angelova MI, Dimitrov DS. Liposome Electroformation. Faraday Discuss Chem Soc. 1986;81: 303–311. doi:10.1039/DC9868100303

7. Kaufmann A, Beier V, Franquelim HG, Wollert T. Molecular mechanism of autophagic membrane-scaffold assembly and disassembly. Cell. 2014;156: 469–481. doi:10.1016/j.cell.2013.12.022

8. Schneider CA, Rasband WS, Eliceiri KW. NIH Image to ImageJ: 25 years of image analysis. Nat Meth. 2012;9: 671–675. doi:10.1038/nmeth.2089
